# Demographic and socio-economic variation in arts engagement across 22 countries: Individual- and country-level factors

**DOI:** 10.1016/j.isci.2026.116637

**Published:** 2026-07-02

**Authors:** Daisy Fancourt, Christos A. Makridis, Ying Chen, Dorota Weziak-Bialowolska, Eric S. Kim, Byron R. Johnson, Tyler J. VanderWeele, Hei Wan Mak

**Affiliations:** 1 Department of Behavioural Science and Health, University College London, London, UK; 2 W. P. Carey School of Business, Arizona State University, Tempe, AZ, USA; 3 Institute for Global Human Flourishing, Baylor University, Waco, TX, USA; 4 Institute for the Future, University of Nicosia, Nicosia, Cyprus; 5 The Gallup Inc., Washington, DC, USA; 6 Human Flourishing Program, Institute for Quantitative Social Science, Harvard University, Cambridge, MA, USA; 7 Department of Epidemiology, Harvard T.H. Chan School of Public Health, Boston, MA, USA; 8 Department of Quantitative Methods & Information Technology, Kozminski University, Warsaw, Poland; 9 Department of Psychology, University of British Columbia, Vancouver, BC, Canada; 10 Lee Kum Sheung Center for Health and Happiness, Harvard T.H. Chan School of Public Health, Boston, MA, USA; 11 Institute for Studies of Religion, Baylor University, Waco, TX, USA

**Keywords:** arts engagement, inequalities, multi-level modeling, meta-analysis, cross-country analysis

## Abstract

Engagement in the arts is a ubiquitous human practice across culture, and interdisciplinary evidence highlights its importance for both individuals and society. However, there is still limited behavioral understanding of global patterns and profiles of arts engagement. To address this, the present study analyzed data from the Global Flourishing Study and reported new behavioral data on arts participation among adults in 22 countries. The study found that the highest arts engagement scores were observed in countries in sub-Saharan Africa, high-income Western societies, and upper-middle-income countries from other cultural spheres, and the differential engagement patterns were explained by both individual- and country-level factors (such as education and the proportion of population aged 65+ years). These findings reveal concerning patterns of inequalities in engagement present at individual and country levels and persistent across the life-course, while highlighting the need for continued data collection on arts engagement as a human behavior.

## Highlights

• Arts engagement rate varied, from 87% in Australia/Nigeria to 13% in Egypt
• Individual- and country-level factors shaped arts engagement
• Findings highlight need for future data to better capture arts engagement as a behavior

## Graphical abstract

**Figure:**
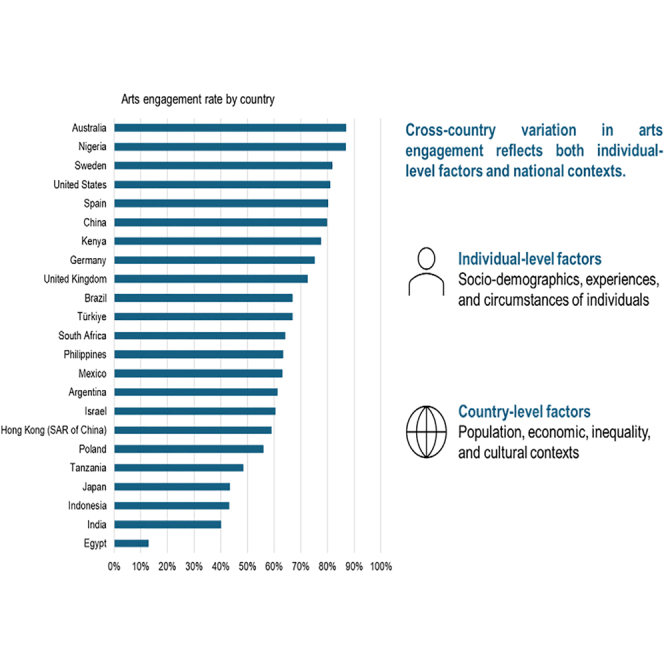

## Teaser

Applied computing; Economics; Sociology

## Introduction

The “arts” encompass a diverse range of human practices ubiquitous in every human culture since the Paleolithic times.^1^ While styles and genres of art vary across cultures, art practices are united in relating to the production/experience of human creativity and imagination and being designed to be appreciated primarily for their beauty, ideas, and/or emotional power.^2^ These practices coalesce into broad categories of participating in or observing dance/movement, literature, media, music, theater/performance, and visual arts/crafts/designs (hereinafter referred to collectively as “the arts”), which are either engaged in as leisure or are embedded into community, cultural, or religious practices.^3^ Evidence from diverse interdisciplinary fields of research highlight the importance and value of the arts to individuals and society, including potential evolutionarily adaptive benefits (e.g., roles in sexual selection, social bonding, neurocognitive development, and communication),^4^^,^^5^ as well as broader societal value for education, criminal justice, society, health, and wellbeing.^6^ As such, the arts are widely accepted as a beneficial human behavior capable of contributing to international targets such as sustainable development goals,^7^^,^^8^^,^^9^ and since 1948, the United Nations (UN) has declared, as part of the Universal Declaration of Human Rights (UDHR), that everyone has the right to “participate in the cultural life of the community” and “to enjoy the arts”.^10^ The majority of UN member states have formally accepted these responsibilities in international treaties associated with the UDHR, and this right has been given legal status in international law by two treaties.^11^

However, while countries’ records on other human rights are held to public scrutiny, engagement with the arts receives comparatively less attention. Indeed, there is limited work exploring patterns and profiles of arts engagement internationally. At individual country levels, there are some examples of relatively in-depth monitoring of arts engagement, such as collection of time-use data and cross-sectional surveys of annual arts participation rates,^12^^,^^13^^,^^14^ but international comparisons largely focus on the European countries.^15^^,^^16^ Part of the reason for this is there are major challenges around conceptualizing and measuring “arts” and gathering and analyzing appropriate cross-national data. In 2009, United Nations Educational, Scientific and Cultural Organization (UNESCO) framework for cultural statistics documented such challenges, including reductionist conceptions of arts engagement, Eurocentric biases in definitions, and inconsistent data available across countries,^17^ but the UNESCO framework also highlighted the importance of such work, stating that governments have a duty to uphold the right to participation in the arts, and this requires monitoring. It further argued that surveys of engagement rates are not always reductionist and can, in fact, generate meaningful and important insights.^17^ In recent years, some opportunities have emerged for analyzing new data on arts engagement being incorporated into routine multi-national surveys. For example, the World Values Survey and OECD datasets such as the Programme for International Student Assessment (PISA) have included questions on aspects of arts engagement such as participation in arts and music community groups and in arts clubs/societies in and outside of secondary school. The questions in these datasets are, admittedly, currently imperfect at capturing the full nuance of a behavior as complex as arts engagement, but they are starting to provide important data demonstrating that inequalities in access to the arts do exist within and between countries, and that participation is not just predicted by individual-level characteristics (so-called “micro” factors) but also by societal (“macro”) factors including economic factors such as country-level income inequality, demographic factors such as net migration rates, and health-related factors such as life expectancy.^18^^,^^19^ This is an advance on some previous cross-European analyses that had largely just focused on micro-level predictors of engagement and highlights the role that governments may have not only in providing the conditions necessary to enable arts engagement^15^^,^^16^ but also in ensuring equitable provision and access to arts and culture through policy, investment, and infrastructure.^20^^,^^21^^,^^22^ In addition, this work advances the understanding of how future survey questions on arts engagement should be designed, with these previous studies also making recommendations for future question adaptations based on learnings developed during the analytical process.^15^^,^^16^ As such, analyzing cross-national data on arts engagement can be valuable in catalyzing awareness and discussions around inequalities in access and supporting methodological development.

The present study was, therefore, conducted in the same vein, making use of new data on arts participation available in a multi-national study of adults living in 22 countries that collectively represent 46% of the global population. A question on arts participation was included in the study as part of the mid-year retention survey. This study question followed key recommendations from the UNESCO framework for cultural statistics, including focusing on only one major type of behavior at once (i.e., just rates of engagement rather than other markers such as ownership of cultural products, cultural expenditure, or cultural meaning), taking a modular approach specifically focusing on active *participation* in arts (i.e., which involves a conscious act of engagement rather than passive exposure, e.g., walking past art in public spaces), assessing frequencies of engagement to differentiate one-off vs. repeated engagement, focusing on mid-term timescales of weekly to yearly (which is recommended as providing the best compromise between providing reliable recall and sufficiently high participation rates to enable socio-demographic analysis), and taking into account rich data on context (geographical, political, social, and cultural).^17^^,^^23^ Our research questions were: (1) is there evidence of meaningful differences in rates of participation in the arts both within and between countries (which we hypothesized there would be); (2) how do specific individual (micro) and country-level (macro) demographic and socio-economic factors predict rates of arts engagement, and whether and if so, how the individual-level associations vary by country (which we hypothesized that the rate of participation would be associated with the factors and that the associations would vary by country); and (3) what insight can be drawn from the data available to inform future monitoring of arts engagement in other large-scale surveys.

## Results

### Descriptive sample

Data were analyzed using the Global Flourishing Study (GFS), which is a longitudinal study of over 200,000 participants in 22 geographically and culturally diverse countries: Argentina, Australia, Brazil, China, Egypt, Germany, Hong Kong (Special Administrative Region of China), India, Indonesia, Israel, Japan, Kenya, Mexico, Nigeria, Philippines, Poland, South Africa, Spain, Sweden, Tanzania, Türkiye, United Kingdom (UK), and United States (US).

In our unimputed sample data (*N* = 127,971), the mean age of respondents was 45.4 years (standard deviation [SD] = 17.0 years); range = 18–75+ years) (Tables S1–S6). Participants were older in Japan (mean = 52.0 years; SD = 16.6 years), US (mean = 51.7 years; SD = 15.7 years), and Sweden (mean = 49.0 years; SD = 17.6 years) and younger in Egypt (mean = 36.9 years; SD = 14.1 years), Nigeria (mean = 34.4 years, SD = 13.2 years), and Tanzania (mean = 36.1 years; SD = 14.9 years). Generally, 51.5% individuals of our sample were female, 62.0% were married or had a partner, 22.7% had a degree, 57.0% were employed, and 45.5% were living in city.

### Prevalence and patterns of engagement

**Figure 1:**
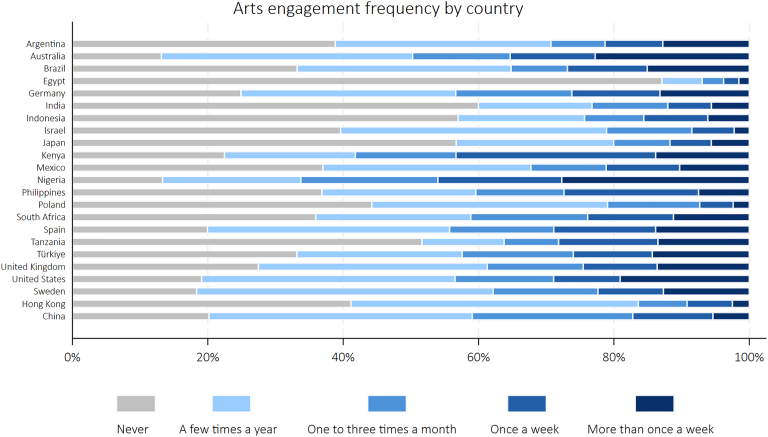
Arts engagement frequency by country Based on unimputed and weighted data.

**Figure 2:**
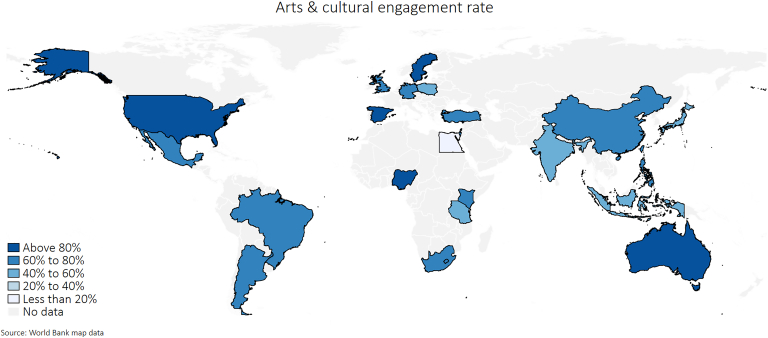
Rates of arts engagement *N* = 131,183 from 22 countries, based on unimputed and weighted data.

In the GFS, participants were asked, “How often do you engage in an arts activity (such as singing, painting, playing a musical instrument, drawing, dancing, textiles, creative writing, photography, or visiting a museum, theater, or concert hall)?” Response items included never, a few times a year, one to three times a month, once a week, and more than once a week (Figure 1 shows the frequency by country). In the main analysis, we collapsed the response items into binary, indicating whether or not the participants had engaged in the arts in the past year. Based on the unimputed and weighted data (*N* = 131,183), people living in Australia, Nigeria, Sweden, US, and Spain had the highest arts engagement rate out of all 22 countries (Figure 2; Table 1). Over 80% of the participants from these countries reported engagement. In contrast, engagement rates were lower in Egypt, India, and Indonesia, in particular, only 12.9% of the participants reporting engagement in Egypt, followed by 40% in India, and 43% in Indonesia.

**Table 1:** Arts engagement rate by country (in table; 131,183 participants from 22 countries, based on unimputed and weighted data)

	Engagement rate
Australia	86.9%
Nigeria	86.7%
Sweden	81.7%
United States	80.9%
Spain	80.1%
China	79.8%
Kenya	77.5%
Germany	75.1%
United Kingdom	72.5%
Türkiye	66.8%
Brazil	66.8%
South Africa	64.1%
Philippines	63.2%
Mexico	63.0%
Argentina	61.2%
Israel	60.4%
Hong Kong	58.9%
Poland	55.8%
Tanzania	48.4%
Japan	43.3%
Indonesia	43.0%
India	40.0%
Egypt	12.9%

### Individual-level factors

To identify key predictors of arts engagement, we looked at both individual-level and country-level factors through two approaches that are based on different assumptions. This helped ascertain whether results were consistent or varied according to which assumption we applied. First, we fitted a two-level logistic model to account for the nested structure of the GFS data (*N* = 131,487 from imputed and weighted data). Second, we performed a meta-analysis on all individual-level predictors, grouping countries by income level: lower-middle income countries (LMICs), upper-middle income countries (UMICs), and higher income countries (HICs).

**Figure 3:**
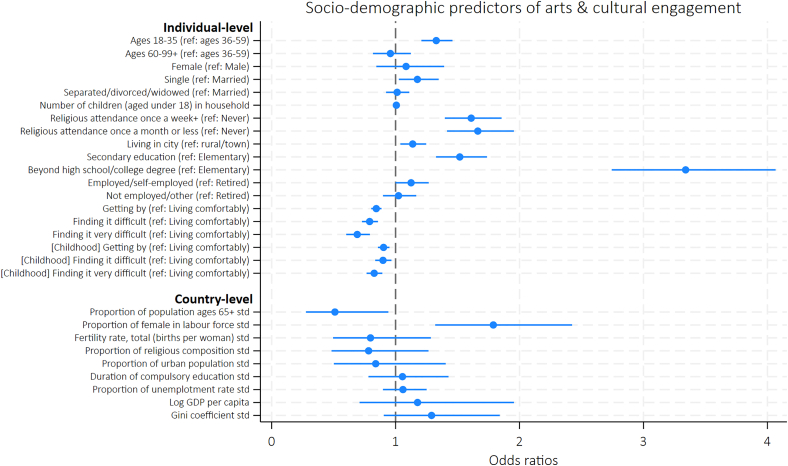
Multilevel modelling estimating the association between individual-level ad country-level socio-economic factors and arts engagement; *N* = 131,487 from 22 countries Individual-level demographic factors included age, gender, marital status, numbers of children (aged under 18 years) in household, attendance frequency of religious services, living area, and education levels. Economic factors are employment status, feelings of their household income, and (childhood) feelings of their family’s household income. Country-level factors included population aged 65+ years (% of total population), females in labor force (% of total labor force), fertility rate: total (births per woman), religious composition %, urban population (% of total population), duration of compulsory education, unemployment rate (% of total labor force), logged GDP per capita, and Gini coefficient. All national predictors (except for GDP per capita) were standardized. The model was run on imputed and weighted data. Odds ratios and 95% CI are presented.

Our analysis shows evidence of an individual-level social gradient in arts engagement (Figure 3; Table S7). Compared to those aged 36–59 years, adults aged 18–35 years were more likely to engage (multilevel modeling [MLM] OR = 1.33, 95% CI = 1.21, 1.46); this finding was present in all categories of country income and in the pooled results from the meta-analysis (pooled effect size = 1.45, 95% CI = 1.31, 1.61) (Figure S1A). Our MLM also found that, compared with those aged 36–59 years, those aged 60–99+ years were no more or less likely to engage; although, this this was not in line with the pooled results, which show that those in the older age group were less likely to engage (pooled effect size = 0.77, 95% CI = 0.67, 0.88), with this association driven by LMICs and UMICs (Figure S1A).

While no association was observed for gender in the MLM or overall meta-analysis, when zooming in on each individual country, males were more likely to engage in LMICs (e.g., India and Tanzania), but females engaged more in HICs (e.g., Australia, Sweden, and US) (Figure S1C). As per marital status, those who were single showed greater arts engagement than couples (MLM OR = 1.18, 95% CI = 1.03, 1.35). This was confirmed in the meta-analysis, primarily driven by LMICs and UMICs (Figure S1D). However, no association was found for the number of children (aged under 18 years) in household (Figure 3; Table S7; Figure S1E). People who engaged in religious activities, whether once a week or more or once a month or less, were more likely to engage in the arts than those who never engaged in religious activities, as confirmed in the meta-analysis (Figure 3; Table S7; Figure S1F). As per living area, engagement rate was higher among those living in city as opposed to those living in rural areas or in towns (MLM: OR = 1.14, 95% CI = 1.04, 1.25; pooled effect size = 1.13, 95% CI = 1.04, 1.23), however, such pattern might be driven by HICs (Figure S1G).

For socio-economic factors, people with higher education levels, those who were living comfortably, and individuals who felt they had lived comfortably when growing up were more likely to engage in the arts, with ORs increasing across ordinal categories (Figure 3; Table S7). These findings were further supported by the pooled results from the meta-analyses. In particular, the associations for education appeared to be most consistent across countries (pooled effect size: 2.10, 95% CI = 1.83, 2.41) (Figure S1H). Those who were employed showed marginally higher engagement than those who were retired, replicated in the meta-analysis, although this appeared to be driven by some HICs, particularly Australia and Germany (Figure S1I). Meta-analyses for feelings about the household income are presented in Figures S1J and S1K.

Predicted probabilities of engagement from the regression models by demographic and socio-economic factors are shown in Table S8. These demonstrate the relative importance of some individual-level factors above others. For example, in Hong Kong, religious attendance was a particularly strong predictor (the probability of engagement was, on average, 141.3 percentage points higher for people who had regular religious attendance), while gender was not (14.4 percentage points higher for women), but this pattern was reversed in Türkiye (13.8 percentage points higher for regular religious attendance but 54.3 percentage points lower for women).

When running MLM analyses again and focusing on repeated participation within a year (i.e., engaging monthly vs. yearly), the social patterning changed (Figure S2). In particular, the socio-economic gradient was much less pronounced. Those who were not employed or were in other employment setting had 10% higher odds of engaging in the arts monthly than those who were retired, whereas those who reported only getting by with household income had 8% lower odds of monthly arts engagement than those who were living comfortably. Further, females had 14% higher odds of engaging in the arts monthly than males. We also observed an opposite pattern for education: individuals with secondary education had 21% lower odds of engaging in the arts monthly than those with up to elementary education. Meanwhile, some associations were consistent with the main analyses, including higher engagement among people who were single, younger, and showed more regular religious attendance.

### Country-level factors

To explore country-level predictors of arts engagement, we merged in country-level data from World Bank Open Data, World Economics, the Pew Research Centre, and Our World in Data. On a country level, prevalence of arts engagement was positively correlated with the proportion of population aged 65+ years (r = 0.22), the proportion of females in labor force (r = 0.70), the proportion of urban population (r = 0.33), duration of compulsory education (r = 0.14), log GDP per capita (r = 0.37), and Gini coefficient (r = 0.20), and it was negatively correlated with fertility rate (total births per woman) (r = −0.13) and religious composition (r = −0.20). The association between engagement and the unemployment rate was very small (r = 0.05) (Figure S3). Given that South Africa, Egypt, and India are potential outliers, we re-run the correlations removing these countries (Figure S4). Results were maintained.

These same country-level factors were also included as potential predictors of arts engagement in the MLM and as potential moderators of the associations between individual-level factors and engagement in the meta-regressions (Figure 3; Table S7). Using MLM, we found that the proportion of population aged 65+ years was negatively associated with arts engagement (OR = 0.51, 95% CI = 0.28, 0.94), and proportion of female in labor force was positively associated with arts engagement (OR = 1.79, 95% CI = 1.32, 2.42), but no other country-level factors were related. When running MLM analyses again and focusing on repeated participation within a year (monthly vs. yearly engagement), proportion of population aged 65+ years and proportion of women in the labor force were no longer related, but countries with higher urban populations showed lower engagement, while countries with higher unemployment rates showed slightly higher engagement (Figure S2).

Using meta-regression to explore the heterogeneity variance in our meta-analysis with the nine country-level factors (Figure S5), our results showed that the proportion of population aged 65+ years (log odds = 0.03, 95% CI = 0.01, 0.04), female labor force participation (log odds = 0.03, 95% CI = 0.01, 0.05), religious composition (log odds = −0.02, 95% CI = −0.03, −0.01), and GDP per capita (log odds = −0.13, 95% CI = −0.20, −0.06) may moderate the association between their relevant individual predictor and engagement. In countries with a greater proportion of aging population, older adults were more likely to engage in the arts compared with younger adults. In countries with higher proportions of women in the labor force (indicative of greater gender equality), women were more likely to engage compared with men. In countries with greater religious composition, individuals who regularly attended religious services were less likely to engage than those who never attended. In countries with higher GDP per capita, individuals who found it very difficult to sustain with their household income were less likely to engage compared with those who lived comfortably. No moderations were found for other factors. Given the limited number of countries and the observational nature of this study, the meta-regression findings should be interpreted as exploratory.

## Discussion

Our results come at an important moment in history as many countries report financial challenges in funding the arts, and there are increasing global calls from artists around the professional challenges they face.^24^^,^^25^^,^^26^ These analyses are presented here with the acknowledgment that accurately phenotyping a behavior as complex as arts engagement is extremely challenging. Nonetheless, this study provides important insights by demonstrating that there are clear differences both within and between countries in self-reported rates of engagement. Rates of engagement varied from 87% in Australia and Nigeria to 43% in Japan and Indonesia, and just 13% in Egypt, echoing similar patterns reported in analyses on other arts behaviors, such as membership of arts-based community groups.^18^ People with the highest arts-engagement scores were found in countries from sub-Saharan Africa (e.g., #2 Nigeria and #7 Kenya), in high-income Western societies spanning several regions (e.g., #1 Australia, #3 Sweden, #4 US, #5 Spain, #8 Germany, and #9 UK), and in upper-middle-income countries from other cultural spheres (e.g., #6 China and the #10 co-leaders Türkiye and Brazil). These results suggest that high levels of arts engagement can emerge across diverse geographic, economic, and cultural contexts.

Demographically, there were evident inequalities according to gender at both micro and macro levels. Women were less likely to engage in middle-income countries but more likely to engage in HICs, with countries with higher gender equality (as approximated by the percentage of women in the labor force) also having higher rates of reported arts engagement. Younger adults showed highest levels of engagement, and engagement among older adults was greater in countries with higher proportion of aging populations. Engagement was consistently higher among people with higher levels of education and greater attendance in religious activities, with religious attendance being particularly important in countries with lower religious compositions and continuing to be an important predictor of repeated arts engagement within a year. We also observed some differences in predictors depending on country income levels. In LMICs and UMICs, people without a partner were less likely to engage. In HICs, engagement was higher in urban areas, although repeated engagement was more common in countries with lower urban populations. There was, however, little meaningful difference by employment status (at micro or macro levels) or household composition. Finally, economically, there were clear inequalities evident, with people who reported financial difficulties, either at the present time or during their own childhood, reporting lower engagement, particularly in countries with higher GDP per capita.

The patterns of arts engagement reported here are not indicators of overarching cultural practices and are presented with important caveats. First, they represent a smaller, more specific focus on rates of arts engagement, which UNESCO acknowledges as just one component of cultural statistics alongside others, such as patterns of attendance, ownership of cultural products, cultural expenditure, cultural motivations and meanings, and so forth.^17^ Even these different components relating to participation sit within a broader ecosystem of cultural creation and dissemination.^17^^,^^27^ As such, the findings presented here should not be used to suggest overall hierarchies of cultural practices between countries. Second, our measure of arts engagement is not perfect. In particular, there were no specific prompts on digital engagement (e.g., animations, online music creation, etc), folk and indigenous practices, storytelling or creative writing or going to libraries, reading, listening to music, or “fringe” activities that may or may not be categorized as arts (e.g., culinary arts such as baking or cooking, horticultural activities such as gardening, and broader creative practices such as tattooing, etc). The question did not specify whether participants had to be engaging in arts as a primary activity (as typically required in time use surveys) so as to enable reporting of arts engagement that might have occurred as secondary to other activities such as religious participation, exercise, or community rituals. Watching arts practices as part of community or religious rituals or festivals typically also involves participation (e.g., watching and partaking in community dance), and therefore, technically, such behaviors may have been reported. However, there was also no prompt on these behaviors, and therefore, responses may have overlooked these embedded artistic practices. Similarly, the question did not distinguish between professional and amateur engagement nor between active vs. receptive engagement, online vs. in person, or individual or social. This lack of differentiation is in line with UNESCO guidelines on using broad categories for reporting, but equally, there was no specific explanation about these factors to participants; therefore, it is unclear whether participants opted not to report certain behaviors.

There was also still evidence of Eurocentric bias in the questions, prompting on viewing arts engagement in formalized settings (e.g., museums and galleries) but not in village or religious settings. However, the critical question is, whether these limitations to the question are likely to have markedly biased responses. Given that the question itself was open and broad (“engaged in an arts activity”) and all suggestions were prompts only, it is likely that diverse activities were still captured in responses. For example, broad terms like “textiles” likely elicited responses on folk practices such as weaving. Additionally, as we were only focusing on relatively infrequent rates of participation (a maximum of once a week), even if individuals did not think that specific activities may have counted, arts behaviors do cluster, so an individual engaging in one activity is highly likely to have also engaged in another that may have been reported.^12^^,^^28^ The data patterns presented are also sensical and align with some anticipated results. For example, it is notable that reported country-level prevalence rates do not suggest higher prevalence rates among WEIRD nations (Western, educated, industrialized, rich, and democratic). This suggests that an arguable Eurocentric bias in the question did not lead to an overall Eurocentric skew in the results. Nonetheless, the results presented here must be interpreted in light of the limitations of the measure used.

Based on our findings here, we highlight some interesting patterns that can form a foundation for future research and policy. UNESCO’s MONDIACULT declaration from 2022 describes the arts as “essential for the genuine development of the individual and society,” and it includes a commitment to addressing barriers in provision and access to the arts.^20^ The resultant framework published in 2024 reinforces the importance of the UN human right on access to the arts and highlights that this access needs to be irrespective of race, gender, or age.^10^ Our findings highlight important and marked inequalities in engagement in the arts within and between countries. In terms of gender inequalities (present at micro and macro levels and in moderation analyses), the striking correlation between a nation’s female labor force participation and arts engagement levels may be understood through at least two lenses. First, from a social capital and network theory lens, as women increasingly participate in the labor force, they often develop broader and more diverse social networks outside the home. This expansion of social capital, with increased “bridging” ties and cross-domain interactions, can create a more fruitful ground for the transmission of cultural ideas, organization of cultural activities, and fostering of cultural life, thus fueling arts engagement.^29^ Second, from the lens of activation threshold, the non-linear curve may indicate that achieving a certain critical mass in female economic labor participation might fundamentally reconfigure societal norms and structures. This could involve a reallocation of collective cultural bandwidth or a shift toward a more even distribution of opportunity costs for discretionary time across genders. Once this threshold is crossed, the system may transition to a new state that is more supportive of widespread cultural participation for all members.

In identifying key socio-economic inequalities in arts engagement, our findings show that wealth and education are important predictors, as previously demonstrated in European studies,^30^ with these effects appearing to operate at micro rather than macro levels. This partly aligns with past work demonstrating associations between arts and music group membership and individual-level but not country-level years of compulsory education; although, in the same study, country-level income inequality had been found to predict overall engagement.^18^ This discrepancy could be because community groups often depend on community infrastructure that may be more available in countries with greater wealth inequalities (e.g., due to greater funding of elite arts institutions); the question in this current analysis included home-based arts engagement, which is less dependent on such infrastructure. Previous European studies had suggested that income and education may play less important roles as determinants of engagement in countries that have, respectively, more generous state arts subsidies or a relatively highly educated labor force,^31^^,^^32^ but this appears not to hold when considering countries from broader geographical and cultural settings. With regards to income, we found that people with lower socio-economic position did engage less with arts, but this was more pronounced in more affluent countries, perhaps because more affluent countries may have greater institutionalization of arts as an elite leisure activity potentially engaged with as part of increasing one’s social status, alongside reduced embedding of the arts within community practices.^33^ With regards to education, Nigeria illustrates the divergence we found, with its remarkably high arts engagement (#2 in our report) despite lower formal tertiary education rate. This is likely driven by Nigeria’s vibrant festival scene, which engages 70% of all citizens in the country and was recently recognized by the British Council as a proxy for vocational colleges and arts centers in developing artistic, managerial, and operational skills within the creative and cultural industries.^34^ This direct engagement in skill transmission through the arts demonstrates how such community-driven infrastructure can operate alongside formal education and infrastructure, thus providing diverse pathways to arts engagement.

It is further concerning that our analyses specifically demonstrate that, independent of current economic circumstances, past economic circumstances during one’s own childhood are predictors of engagement. This echoes work previously carried out in individual countries,^13^^,^^14^^,^^35^ and highlights the role of intangible cultural “capital” (e.g., knowledge, skills, and values associated with arts engagement) passed down generations in determining individuals’ behavioral patterns of engagement.^33^ This “early-scarcity scar” becomes clearer when viewed through three complementary lenses. (1) From a developmental calibration perspective, children raised in resource-scarce environments develop shorter time horizons and prefer immediate, lower-risk pay-offs.^36^ They may perceive a poorer return on investment for arts activities, and this perception can carry into adulthood. (2) From a cognitive bandwidth perspective, poverty absorbs cognitive bandwidth and tunnels attention toward budgeting or managing bills.^37^ Thus, there is less cognitive fuel available for creative play. Arts are not ignored because they lack value, but they are de-prioritized because taking part in them requires extra mental space that chronic financial worry has already filled. (3) From the lens of cumulative-stress cascades, resource constraints cascade into chronic time scarcity, elevated allostatic load, and reduced self-efficacy, each compounding the arts engagement gap.^38^ However, it is notable that when looking at repeated (rather than one-off) arts engagement, socio-economic factors became less strong predictors, which could suggest that finances and education are key enablers of initial engagement, but if arts behaviors can become embedded within day-to-day life, they are not so dependent on financial resources.

These collective findings warn against overly simplistic policy or intervention solutions to address the inequalities presented and instead suggest the importance of considering what cross-generational socio-economic inequalities in participation rates reveal about underlying barriers to the arts. Previous theoretical models such as the RADIANCE framework of determinants of arts engagement have demonstrated that, within individual countries, barriers to the arts can operate at individual, community, and societal levels.^39^ To achieve meaningful and sustained change, action needs to be taken at multiple levels simultaneously (e.g., working to increase motivations among individuals to engage as well as identifying and addressing wider community and societal barriers).^39^ Therefore, an important next step is to understand more about the nuanced patterns of socio-economic inequalities reported in this paper. Furthermore, even though some of the inequalities in engagement were similar across countries, it is also important not to assume that the same barriers are responsible for these inequalities. As theoretical frameworks, such as RADIANCE, demonstrate, specific barriers to arts engagement depend on the cultural policy and socio-economic conditions of respective countries. For cultural policy alone, countries vary enormously in the approaches they take, from weak to strong state intervention, and whether individuals are seen as passive recipients of arts vs. active participants or generators.^40^ Additionally, the broader political and socio-economic conditions of countries will guide and constrain the kinds of interventions or policy initiatives that could be taken. Furthermore, our analyses also revealed how inequalities in access were not just related to micro-level factors but also related to, and moderated by, macro-level ones. Consequently, the international lens of this paper provides an important context to interpreting the country-level findings, suggesting that addressing inequalities requires consideration of both national and local contexts to avoid leading to overly narrow proposed “solutions”.

The analysis of the data presented here also informs future thinking on how more comprehensive, culturally sensitive measures can be developed for future surveys. UNESCO’s recent MONDIACULT declaration explicitly calls for work “strengthen[ing] and develop[ing] instruments and mechanisms for the integrated analysis, monitoring, and measurement of culture and its impact on sustainable development […] through the development of conceptual studies on the impact of culture in all its dimensions.”^20^ We support this call and recommend that future survey questions are developed as part of international consortia of artists and researchers in different countries. In particular, based on the learnings gleaned from the analyses presented here, we argue that it is important that future surveys include questions that more explicitly prompt on artistic practices that might be embedded within religious, community, or folk rituals or traditions in order to confirm whether the socio-economic inequalities reported here are genuinely present in these other practices too. More recent UNESCO guidelines published since the data here were collected provide more advanced conceptual and methodological frameworks, including classifications guides for the development of such measures, which will prove valuable resources moving forward.^27^ In addition, we echo UNESCO’s recommendation that arts participation data be collected regularly (either repeat cross-sectionally or longitudinally), rather than one-off, to enable monitoring of time changes in participation rates and the evaluation of specific interventions such as new cultural policies. The overarching goal should be to iteratively test and hone culturally sensitive survey items on arts engagement that can eventually be incorporated into national and international surveillance systems such as the World Health Organization (WHO) Global Health Observatory, enabling the monitoring of arts engagement alongside other health-related behaviors such as physical activity, substance use, and diet.

### Limitations of the study

This study had a number of strengths, including its large-scale samples including quota-representative samples from 22 countries representing nearly the half the world’s population. Data were gathered using pre-harmonized questions, in local languages, across a short (aligned) time period. The data included rich socio-demographic and economic data and our analyses additionally incorporated rich country-level data from reputable sources. However, there are a number of additional limitations to this study, beyond those listed here already. In particular, we can only report correlations rather than causal estimates. As another limitation, some of our analyses included MLMs, which assume measurement invariance across languages and cultures which may not hold. However, this is a common limitation with multi-national multi-level analyses, and we deliberately incorporated two different statistical approaches with different assumptions in order to ascertain how convergent the conclusions were. In actuality, our findings were very similar when using MLM vs. standard meta-analytic approaches, which increases confidence in our results. That said, bias may still occur if survey items were interpreted differently across countries or if there was construct misalignment across contexts. In addition, we do not have more granular measures of the type of arts exposure, so we cannot separately identify the effects of different activities. Further, all measures in the study were self-report, which provides a potential source of recall or social desirability bias. We also chose example measures of each socio-demographic factor we were interested in at micro and macro levels, based on questions that have been widely used in past studies, are regarded as having good data quality, and have been shown to perform well comparatively between countries, but that does not mean that different measures would not reveal different findings. This reinforces the need for further studies to enable longer-term triangulation and comparative assessment of findings. We were also unable to compare other types of gender due to a very small sample size. While this study explored both micro- and macro-level socio-economic indicators, more specific work on specific clusters of factors (e.g., access to the art, opportunities for engagement, or structural barriers) is also encouraged to identify “ecological niches” of key factors that converge together to form strong predictors of arts engagement.

In conclusion, this study provides important demonstration that arts engagement varies substantially within and between countries, with concerning patterns of inequalities in engagement present at individual and country levels and persistent across the life-course. It is only through analysis of data that we gain a fully nuanced understanding of how future improved data can be captured. The analyses presented here thus also provide new insights and recommendations for how future data can be collected to more accurately phenotype arts engagement as a behavior.

## Resource availability

### Lead contact

Further information and requests for resources should be directed to and will be fulfilled by the lead contact, Prof. Daisy Fancourt (d.fancourt@ucl.ac.uk).

### Materials availability

This study did not generate any new materials.

### Data and code availability

• The Global Flourishing Study data are publicly available upon preregistration through the Center for Open Sciences (COS; Johnson et al., 2024, https://osf.io/3jtz8/).
• Analytical plans were pre-registered at https://osf.io/t5h79/overview.
• All codes for the analysis can be found at https://osf.io/xa3h8/overview.

## Acknowledgments

This work was carried out as part of the EpiArts Lab, a National Endowment for the Arts Research Lab at the 10.13039/100007698University of Florida, which is supported in part by an award from the 10.13039/100000193National Endowment for the Arts (1862896–38-C-20). The opinions expressed are those of the authors and do not represent the views of the National Endowment for the Arts Office of Research & Analysis or the National Endowment for the Arts. The National Endowment for the Arts does not guarantee the accuracy or completeness of the information included in this material and is not responsible for any consequences of its use. The EpiArts Lab is also supported by the 10.13039/100007698University of Florida, Americans for the Arts, the Pabst Steinmetz Foundation, and 10.13039/100015283Bloomberg Philanthropies. In addition, H.W.M. was supported by the Jameel Arts & Health Lab Global Population Health Fellowship and the 10.13039/100014013UK Research and Innovation (MR/Y01068X/1). The Global Flourishing Study was supported by funding from the 10.13039/100000925John Templeton Foundation (grant no. 61665, to B.R.J. and T.J.V.), 10.13039/501100013437Templeton Religion Trust (grant no. 1308, to B.R.J. and T.J.V.), 10.13039/501100011730Templeton World Charity Foundation (grant no. 0605, to B.R.J. and T.J.V.), Well-Being for Planet Earth Foundation, 10.13039/100001614Fetzer Institute (grant no. 4354, to B.R.J. and T.J.V.), Well Being Trust, Paul L. Foster Family Foundation, and the David and Carol Myers Foundation. The opinions expressed in this publication are those of the authors and do not necessarily reflect the views of these organizations.

## Author contributions

D.F. and H.W.M. conceived the study; H.W.M. analyzed the data; D.F. and H.W.M. drafted the manuscript. All authors edited and approved the manuscript.

## Declaration of interests

The authors declare no competing interests.

## STAR★Methods

### Key resources table

**Table:** 

REAGENT or RESOURCE	SOURCE	IDENTIFIER
**Deposited data**		

Dataset	Global Flourishing Study	https://globalflourishingstudy.com/
Pre-registration	Open Science Forum (OSF)	https://osf.io/t5h79/overview
Code	Open Science Forum (OSF)	https://osf.io/xa3h8/overview

### Method details

#### The Global Flourishing Study (GFS)

GFS is a longitudinal study of over 200,000 participants in 22 geographically and culturally diverse countries, with nationally representative sampling. These include Argentina, Australia, Brazil, China, Egypt, Germany, Hong Kong (Special Administrative Region of China), India, Indonesia, Israel, Japan, Kenya, Mexico, Nigeria, Philippines, Poland, South Africa, Spain, Sweden, Tanzania, Türkiye, United Kingdom (UK), and United States (US). Some countries used both or either probability-based and non-probability based sampling approaches for recruitment, whereas the US, Sweden and Hong Kong (S.A.R. of China) only used existing web panels with set quotas for age, gender, region and education to ensure adequate representation of the population.^41^ GFS collects rich data to understand various aspects of flourishing and its determinants, and contains numerous socio-demographic, economic, political, religious, personality, lifestyle, community, health and wellbeing variables. Data were also collected on retrospective assessments of childhood experiences. The data are publicly available upon preregistration through the Center for Open Sciences (COS; Johnson et al., 2024, https://osf.io/3jtz8/).

This present study analyzed data from Wave 1 (2022-23) and mid-year retention data (Nov 2023-Nov 2024) which comprised data on arts engagement. The mid-year survey was not administered in a few countries for various reasons such as cost implications and the utility of retention activity. These countries included China, Hong Kong (S.A.R. of China), Japan, Israel, Sweden and the US. For these countries, the substantive mid-year survey questions (including arts engagement question) were included in the second annual survey to ensure all respondents had an opportunity to answer these items (Jan 2024-Dec 2024). Of all Wave 1 respondents, 131,487 individuals (63.2%) answered the mid-year or Wave 2 interview. Of these, 131,183 completed the arts measure and 126,932 provided complete data across measures used in our present study. Ethical approval was granted by the institutional review boards at Baylor University and Gallup, and all participants provided informed consent.

Analytical plans were pre-registered https://osf.io/t5h79/overview. Analyses proceeded according to the pre-registration, with two exceptions. First, we substituted log GDP per capita at purchasing power parity for log GDP per capita as the former focuses on quality of life/purchasing power, whereas we wanted to focus on economic development. Second, in the meta-analyses using multiple imputations, marital status and education levels were dichotomized into married vs not married and with degree vs without degree to simplify the models as there were issues with non-convergence when using our original categorical approach.

#### Measures

##### Arts engagement

Arts engagement was measured via a single-item measure asking *How often do you engage in an arts activity (such as singing, painting, playing a musical instrument, drawing, dancing, textiles, creative writing, photography, or visiting a museum, theatre, or concert hall)?* Response items included never, a few times a year, one to three times a month, once a week, and more than once a week. This level of frequency enabled differentiation between one-off and repeated engagement, while avoiding assessments of more frequent amounts of engagement that could be subject to recall bias or confusion (such as requiring participants to identify the number of hours of engagement).

We first reported overall frequencies of engagement for each country (Figure 1), then generated a binary indicator, indicating whether or not they engaged in the arts in the past year. We then generated a further binary indicator as a sensitivity analysis to differentiate between yearly engagement and monthly or more engagement.

##### Factors of engagement

A rich set of both individual and country-level demographic and socio-economic factors measured at Wave 1 was considered to understand how arts engagement varied across these factors.

*Individual/micro-level factors:* For individual demographic factors, we considered age (ages 18-35, ages 36-59, ages 60-99+), gender (female vs male), marital status (single, married/have a domestic partner, separated/divorced/widowed), number of children aged under 18 in the household, religious attendance frequency (never, once a month or less, once a week or more), and living area (rural/farm/small town/village vs a large city/suburb of a large city). For individual socio-economic factors, we considered education levels (up to elementary education, secondary education, beyond high school/college degree), employment status (employed/self-employed, retired, not employed/other), feelings about household income (living comfortably, getting by, finding it difficult, finding it very difficult), and feelings about family’s household income when aged 12 (living comfortably, getting by, finding it difficult, finding it very difficult).

*Country/macro-level factors:* For country-level demographic factors, we merged data from World Bank Open Data, World Economics, the Pew Research Centre and Our World in Data. We considered population ages 65 and above (% of total population),^42^ proportion of female in the labor force (% of total labor force),^42^ fertility rate, total (births per woman),^42^ religious composition % (i.e. the proportion of people who identified with any religion),^43^ and urban population (% of total population).^42^ For country-level socio-economic factors, we considered duration of compulsory education,^44^^,^^45^ unemployment rate (% of total labor force),^42^ log GDP per capita (current US$),^46^ and Gini coefficient for income inequality.^47^

### Quantification and statistical analysis

To identify key predictors of arts engagement, we used two approaches. First, we fitted a two-level logistic model to account for the nested structure of the GFS data, as people living in the same country were likely to have similar experiences, lifestyles and behaviors to each other than other living in another country. The model allowed the intercept to vary. All nine country-level predictors were entered simultaneously; the risk of multicollinearity was minimal (VIF=3.9). The intraclass correlation coefficient indicated that 8.5% of the variance in the outcome was attributed to differences between country, supporting the use of a multilevel logistic regression model. Odds ratios and 95% confidence intervals (CIs) are reported to present the direction and magnitude of the association between demographic and socio-economic predictors and engagement. Missing data on all variables were imputed using multiple imputations by chained equations and five imputed datasets were used. Multiple imputation was conducted under the assumption that data were missing at random, meaning that the missingness may be related to observed characteristics included in the imputation model. All individual demographic and socio-economic predictors, the outcome variable, and a sampling weight were used for the imputation. Given that all these variables were measured across all countries and that the analysis was performed on the pooled sample, the imputation process was conducted with the full data. Results from the imputation diagnostics indicated that the relative increases in variance (RVI) was low, suggesting that additional variance introduced by missing data was small (Table S9). The low values of the fraction of missing information (FMI) similarly indicated that only a small proportion of the total sampling variance was attributed to missing data. Together with the high relative efficiency (RE), which was consistently above 99% across variables, these results suggest that using five imputations was adequate.

Following these analyses, we performed a meta-analysis on all individual-level predictors. We first analyzed the associations between predictors and engagement for each country using logistic regression. To match with the standard statistical modelling procedure, a different multiple imputation approach was conducted. Within-country imputation approach was used to ensure the accuracy of the imputation models. All individual socio-economic predictors, the outcome variable, and a sampling weight were used for the imputation. Marital status and education levels were dichotomized into married vs not married and with degree vs without degree due to model non-convergence. Country specific estimates (odds ratios and 95%CI) were then pooled using a random-effects meta-analysis, as the effect sizes were expected to vary across countries, to estimate the overall effect sizes of each predictor across countries. In addition, we also conducted a subgroup analysis by country-income category subgroup (namely lower-middle income countries [LMICs], upper-middle income countries [UMICs], and higher income countries [HICs], defined according to the World Bank classification)^48^ to explore whether the differences in effect sizes might have attributed to country income. Heterogeneity was examined by computing I^2^, H^2^ and T^2^ statistics. I^2^ is the percentage of variability in the effect size that is caused by between-country heterogeneity, rather than by sampling error, with values of 25%, 50%, and 75% indicating low, moderate, and high, respectively.^49^ The H^2^ statistics describes the ratio of the observed variation and the expected variance due to sampling error, with larger values indicating greater heterogeneity.^50^ The T^2^ statistics is the variance in proportions across countries and is an indicator of cross-national heterogeneity, with larger values indicating greater heterogeneity.^51^^,^^52^

We further conducted exploratory meta-regression to examine the heterogeneity variance in our meta-analysis using the nine country-level factors. Given the limited number of countries, we explored each country-level factor individually. Each country-level factor was matched with a relevant individual factor: the proportion of population aged 65+ in a country was matched with individual age, the proportion of female in the labor force with individual gender, fertility rate with the number of children in the household, religious composition % with individual religious attendance, urban population with individual living area, duration of compulsory education with individual education level, the unemployment rate with individual employment status, GDP per capita with feelings about household income, and Gini coefficient with feelings about household income when growing up.

Whilst both multilevel modelling (MLM) and meta-analysis are based on different assumptions, including the former assuming measurement invariance across languages and cultures and the latter treating each country as an individual entity, both could be argued to be appropriate in this context. So we deliberately conducted both analyses in parallel, in order to ascertain whether results were consistent or varied according to which assumption we applied.

Longitudinal mid-year sampling weights provided by GFS were applied throughout to account for the complex survey design. All codes for the analysis can be found at https://osf.io/xa3h8/overview. Analyses were carried out in Stata v18.
