# Supplementary material for: Demographic and socio-economic variation in arts engagement across 22 countries: Individual- and country-level factors
Source: iScience. 2026 Jul 2;29(7):116637. doi: 10.1016/j.isci.2026.116637 (PMC13355013; doi:10.1016/j.isci.2026.116637)
Supplement: Document S1. Figures S1–S5 and Tables S1–S9 [file mmc1.pdf]

iScience, Volume *29*

Supplemental information

Demographic and socio-economic variation in arts engagement across 22 countries: Individual- and country-level factors

Daisy Fancourt, Christos A. Makridis, Ying Chen, Dorota Weziak-Bialowolska, Eric S. Kim, Byron R. Johnson, Tyler J. VanderWeele, and Hei Wan Mak

Supplementary Materials

Table S1: Average age and standard deviation (SD) by arts engagement status amongst the analytical subsample. N=127,971; based on unimputed and weighted data (participants with missing data on any individual-level sociodemographic factors were excluded).

	**Overall**		**Engaged**		**Not engaged**	
	Mean	(SD)	Mean	(SD)	Mean	(SD)
Japan	52.0	(16.6)	51.8	(17.7)	52.2	(15.8)
United States	51.7	(15.7)	51.0	(15.8)	54.7	(14.7)
Sweden	49.0	(17.6)	49.3	(17.8)	47.6	(16.7)
United Kingdom	50.0	(16.3)	48.0	(16.0)	55.4	(15.6)
Australia	48.9	(17.1)	47.8	(17.0)	56.1	(15.8)
Germany	49.2	(16.8)	47.4	(16.9)	54.7	(15.3)
Spain	46.7	(14.8)	45.9	(14.9)	50.3	(14.0)
Hong Kong	47.9	(14.9)	45.9	(15.2)	50.8	(14.0)
Poland	46.6	(16.4)	45.5	(16.8)	48.0	(15.7)
**Total**	**45.4**	**(17.0)**	**44.9**	**(17.2)**	**46.3**	**(16.7)**
China	44.8	(15.3)	44.3	(15.3)	46.4	(15.3)
Israel	44.0	(17.7)	43.4	(17.6)	44.9	(17.9)
Brazil	42.6	(16.0)	41.3	(16.0)	45.1	(15.6)
Argentina	43.2	(16.2)	41.1	(15.8)	46.6	(16.4)
Türkiye	42.0	(16.3)	38.3	(15.1)	49.5	(16.0)
Mexico	40.8	(15.8)	37.7	(14.8)	46.2	(16.1)
Philippines	38.7	(15.2)	37.7	(15.1)	40.5	(15.1)
South Africa	38.8	(15.0)	37.0	(13.9)	42.1	(16.3)
India	37.6	(14.8)	35.9	(14.4)	38.7	(14.9)
Indonesia	38.9	(13.9)	35.2	(13.8)	41.6	(13.4)
Kenya	35.4	(14.7)	34.2	(14.0)	39.5	(16.3)
Tanzania	36.1	(14.9)	34.0	(14.2)	38.1	(15.3)
Nigeria	34.4	(13.2)	33.8	(12.6)	38.1	(15.5)
Egypt	36.9	(14.1)	30.7	(11.9)	37.8	(14.2)

Table S2: The proportion of female and male gender by arts engagement status amongst the analytical subsample. N=127, 971; based on unimputed and weighted data (participants with missing data on any individual-level sociodemographic factors were excluded).

	Overall		Engaged		Not engaged	
	**Female**	**Male**	**Female**	**Male**	**Female**	**Male**
Japan	52.6%	47.4%	58.2%	41.8%	48.2%	51.8%
Hong Kong	55.7%	44.3%	56.3%	43.7%	55.0%	45.0%
Poland	53.0%	47.0%	55.7%	44.3%	49.6%	50.4%
Israel	51.1%	48.9%	54.8%	45.2%	45.5%	54.5%
United States	52.3%	47.7%	54.7%	45.3%	42.5%	57.5%
Sweden	50.6%	49.4%	53.6%	46.4%	37.1%	62.9%
South Africa	52.3%	47.7%	53.2%	46.8%	50.7%	49.3%
Australia	50.3%	49.7%	52.7%	47.3%	34.6%	65.4%
Germany	51.6%	48.4%	52.7%	47.3%	48.2%	51.8%
United Kingdom	52.5%	47.5%	52.6%	47.4%	52.3%	47.7%
**Total**	**51.5%**	**48.5%**	**51.9%**	**48.1%**	**50.6%**	**49.4%**
Spain	50.0%	50.0%	51.4%	48.6%	44.8%	55.2%
Mexico	52.6%	47.4%	49.9%	50.1%	57.4%	42.6%
Argentina	52.2%	47.8%	49.8%	50.2%	56.0%	44.0%
Brazil	51.9%	48.1%	49.4%	50.6%	57.1%	42.9%
China	49.3%	50.7%	49.4%	50.6%	49.2%	50.8%
Nigeria	49.9%	50.1%	48.8%	51.2%	57.1%	42.9%
Kenya	51.2%	48.8%	48.6%	51.4%	60.0%	40.0%
Philippines	52.0%	48.0%	48.6%	51.4%	57.7%	42.3%
Türkiye	50.0%	50.0%	46.7%	53.3%	56.4%	43.6%
Tanzania	52.9%	47.1%	44.4%	55.6%	60.9%	39.1%
Indonesia	50.0%	50.0%	42.0%	58.0%	55.9%	44.1%
India	48.4%	51.6%	40.5%	59.5%	53.7%	46.3%
Egypt	48.9%	51.1%	35.2%	64.8%	50.9%	49.1%

Table S3: The proportion of married/have a partner and not married by arts engagement status amongst the analytical subsample. N=127, 971; based on unimputed and weighted data (participants with missing data on any individual-level sociodemographic factors were excluded).

	**Overall**		**Engaged**		**Not engaged**	
	**Married or have a partner**	**Not married**	**Married or have a partner**	**Not married**	**Married or have a partner**	**Not married**
India	78.4%	21.6%	73.7%	26.3%	81.6%	18.4%
China	73.8%	26.2%	73.5%	26.5%	75.0%	25.0%
Hong Kong	61.4%	38.6%	64.9%	35.1%	56.5%	43.5%
Sweden	63.3%	36.7%	63.3%	36.7%	63.2%	36.8%
Poland	65.0%	35.0%	63.2%	36.8%	67.3%	32.7%
United States	62.5%	37.5%	63.1%	36.9%	59.8%	40.2%
Philippines	66.2%	33.8%	63.0%	37.0%	71.7%	28.3%
**Total**	**62.0%**	**38.0%**	**60.5%**	**39.5%**	**64.9%**	**35.1%**
Israel	59.8%	40.2%	59.4%	40.6%	60.4%	39.6%
Türkiye	64.1%	35.9%	59.2%	40.8%	73.9%	26.1%
United Kingdom	58.0%	42.0%	58.7%	41.3%	56.1%	43.9%
Nigeria	58.5%	41.5%	58.4%	41.6%	58.8%	41.2%
Australia	58.0%	42.0%	58.2%	41.8%	56.9%	43.1%
Tanzania	63.8%	36.2%	58.2%	41.8%	69.1%	30.9%
Spain	58.6%	41.4%	57.9%	42.1%	61.5%	38.5%
Indonesia	67.9%	32.1%	56.5%	43.5%	76.4%	23.6%
Kenya	58.1%	41.9%	56.5%	43.5%	63.4%	36.6%
Japan	59.0%	41.0%	56.2%	43.8%	61.2%	38.8%
Germany	56.5%	43.5%	55.9%	44.1%	58.4%	41.6%
Mexico	56.7%	43.3%	55.0%	45.0%	59.8%	40.2%
Brazil	50.4%	49.6%	50.4%	49.6%	50.3%	49.7%
Egypt	68.5%	31.5%	48.0%	52.0%	71.5%	28.5%
Argentina	46.1%	53.9%	45.7%	54.3%	46.8%	53.2%
South Africa	30.9%	69.1%	29.0%	71.0%	34.2%	65.8%

Table S4: The proportion of with degree and no degree by arts engagement status amongst the analytical subsample. N=127, 971; based on unimputed and weighted data (participants with missing data on any individual-level sociodemographic factors were excluded).

	Overall		Engaged		Not engaged	
	**With degree**	**No degree**	**With degree**	**No degree**	**With degree**	**No degree**
Israel	50.0%	50.0%	58.7%	41.3%	36.9%	63.1%
United States	40.9%	59.1%	45.4%	54.6%	21.8%	78.2%
United Kingdom	36.6%	63.4%	44.9%	55.1%	14.3%	85.7%
Australia	33.5%	66.5%	36.7%	63.3%	12.4%	87.6%
Germany	31.6%	68.4%	36.5%	63.5%	16.7%	83.3%
Poland	28.0%	72.0%	33.9%	66.1%	20.5%	79.5%
Japan	24.2%	75.8%	32.5%	67.5%	17.8%	82.2%
Sweden	25.7%	74.3%	28.5%	71.5%	13.1%	86.9%
**Total**	**22.7%**	**77.3%**	**28.0%**	**72.0%**	**12.1%**	**87.9%**
Mexico	21.0%	79.0%	27.1%	72.9%	10.4%	89.6%
Hong Kong	21.5%	78.5%	26.6%	73.4%	14.0%	86.0%
Türkiye	19.4%	80.6%	26.5%	73.5%	5.4%	94.6%
Spain	23.0%	77.0%	26.1%	73.9%	10.8%	89.2%
Egypt	12.5%	87.5%	25.3%	74.7%	10.7%	89.3%
Brazil	17.0%	83.0%	20.0%	80.0%	10.7%	89.3%
Argentina	9.9%	90.1%	13.6%	86.4%	4.2%	95.8%
China	9.4%	90.6%	10.1%	89.9%	6.6%	93.4%
South Africa	5.7%	94.3%	7.2%	92.8%	3.1%	96.9%
Kenya	6.4%	93.6%	7.1%	92.9%	3.8%	96.2%
Indonesia	4.3%	95.7%	7.0%	93.0%	2.3%	97.7%
India	5.1%	94.9%	6.6%	93.4%	4.0%	96.0%
Philippines	6.3%	93.7%	6.4%	93.6%	6.1%	93.9%
Nigeria	1.7%	98.3%	1.7%	98.3%	1.5%	98.5%
Tanzania	1.0%	99.0%	1.5%	98.5%	0.6%	99.4%

Table S5: The proportion of employed and retired or not employed by arts engagement status amongst the analytical subsample. N=127, 971; based on unimputed and weighted data (participants with missing data on any individual-level sociodemographic factors were excluded).

	Overall		Engaged		Not engaged	
	Employed	Retired or not employed	Employed	Retired or not employed	Employed	Retired or not employed
Hong Kong	74.3%	25.7%	77.3%	22.7%	70.0%	30.0%
United Kingdom	63.7%	36.3%	69.3%	30.7%	48.7%	51.3%
Argentina	62.0%	38.0%	67.6%	32.4%	53.3%	46.7%
Nigeria	66.2%	33.8%	66.0%	34.0%	67.5%	32.5%
China	64.0%	36.0%	65.7%	34.3%	57.2%	42.8%
Israel	58.7%	41.3%	64.7%	35.3%	49.6%	50.4%
Germany	61.4%	38.6%	64.4%	35.6%	52.3%	47.7%
Australia	60.1%	39.9%	62.3%	37.7%	45.7%	54.3%
United States	60.0%	40.0%	61.2%	38.8%	55.0%	45.0%
Japan	62.5%	37.5%	61.0%	39.0%	63.6%	36.4%
Poland	60.9%	39.1%	60.4%	39.6%	61.5%	38.5%
Spain	57.3%	42.7%	60.0%	40.0%	46.5%	53.5%
Sweden	60.6%	39.4%	59.7%	40.3%	64.5%	35.5%
**Total**	**57.0%**	**43.0%**	**58.9%**	**41.1%**	**53.4%**	**46.6%**
Tanzania	55.8%	44.2%	58.1%	41.9%	53.6%	46.4%
Mexico	51.6%	48.4%	56.5%	43.5%	43.1%	56.9%
Indonesia	51.8%	48.2%	54.2%	45.8%	50.0%	50.0%
Egypt	45.3%	54.7%	52.3%	47.7%	44.3%	55.7%
Brazil	48.9%	51.1%	51.9%	48.1%	42.7%	57.3%
Philippines	50.1%	49.9%	51.7%	48.3%	47.3%	52.7%
Türkiye	44.4%	55.6%	49.2%	50.8%	34.7%	65.3%
India	47.6%	52.4%	48.8%	51.2%	46.8%	53.2%
Kenya	42.9%	57.1%	43.2%	56.8%	41.7%	58.3%
South Africa	34.7%	65.3%	34.9%	65.1%	34.4%	65.6%

Table S6: The proportion of living in city and living in rural or town by arts engagement status amongst the analytical subsample. N=127, 971; based on unimputed and weighted data (participants with missing data on any individual-level sociodemographic factors were excluded).

	Overall		Engaged		Not engaged	
	**Living in city**	**Living in rural or town**	**Living in city**	**Living in rural or town**	**Living in city**	**Living in rural or town**
Hong Kong	91.5%	8.5%	92.7%	7.3%	89.7%	10.3%
Türkiye	73.9%	26.1%	74.8%	25.2%	71.9%	28.1%
Australia	72.1%	27.9%	73.7%	26.3%	61.3%	38.7%
Israel	66.2%	33.8%	66.8%	33.2%	65.2%	34.8%
Argentina	64.8%	35.2%	65.0%	35.0%	64.5%	35.5%
Brazil	61.1%	38.9%	63.3%	36.7%	56.6%	43.4%
United States	59.3%	40.7%	60.7%	39.3%	53.3%	46.7%
China	58.6%	41.4%	58.4%	41.6%	59.5%	40.5%
Spain	52.4%	47.6%	53.5%	46.5%	48.1%	51.9%
Mexico	46.5%	53.5%	49.6%	50.4%	41.0%	59.0%
Japan	44.0%	56.0%	48.9%	51.1%	40.2%	59.8%
**Total**	**45.5%**	**54.5%**	**48.7%**	**51.3%**	**39.2%**	**60.8%**
Sweden	47.0%	53.0%	48.6%	51.4%	39.6%	60.4%
Germany	46.6%	53.4%	47.8%	52.2%	43.0%	57.0%
United Kingdom	44.7%	55.3%	45.8%	54.2%	41.8%	58.2%
Poland	37.4%	62.6%	45.1%	54.9%	27.7%	72.3%
Nigeria	41.1%	58.9%	41.9%	58.1%	36.3%	63.7%
Philippines	41.4%	58.6%	40.6%	59.4%	42.7%	57.3%
South Africa	34.3%	65.7%	36.0%	64.0%	31.3%	68.7%
Egypt	34.0%	66.0%	31.5%	68.5%	34.4%	65.6%
Indonesia	27.6%	72.4%	28.2%	71.8%	27.2%	72.8%
Tanzania	25.8%	74.2%	25.4%	74.6%	26.1%	73.9%
India	24.1%	75.9%	22.2%	77.8%	25.4%	74.6%
Kenya	11.8%	88.2%	12.3%	87.7%	9.8%	90.2%

Table S7: Results from multilevel logistic modelling for associations between individual-level and country-level socioeconomic factors and arts engagement (based on imputed and weighted data).

	OR	(95%CI)
Fixed part		
Individual-level		
Ages 18-35 (ref ages 36-59)	**1.33**	**(1.21 - 1.46)**
Ages 60-99+ (ref ages 36-59)	0.96	(0.82 - 1.12)
Female (ref male)	1.08	(0.84 - 1.39)
Marital status: Single (ref married)	**1.18**	**(1.03 - 1.35)**
Marital status: Separated/divorced/widowed (ref married)	1.01	(0.92 - 1.11)
Number of children aged under 18 in household	1.01	(0.98 - 1.03)
Frequency of religious attendance: Once a week or more (ref never)	**1.61**	**(1.40 - 1.86)**
Frequency of religious attendance: Once a month or less (ref never)	**1.66**	**(1.42 - 1.95)**
Living in city (ref rural/town)	**1.14**	**(1.04 - 1.25)**
Education: Secondary education (ref up to elementary)	**1.52**	**(1.33 - 1.74)**
Education: Beyond high school or college degree (ref up to elementary)	**3.34**	**(2.74 - 4.07)**
Employment status: Employed or self-employed (ref retired)	1.13	(1.00 - 1.27)
Employment status: Not employed or other (ref retired)	1.02	(0.90 - 1.17)
Feelings about household income: Getting by (ref living comfortably)	**0.84**	**(0.80 - 0.89)**
Feelings about household income: Finding it difficult (ref living comfortably)	**0.79**	**(0.73 - 0.86)**
Feelings about household income: Finding it very difficult (ref living comfortably)	**0.69**	**(0.60 - 0.79)**
[Childhood] Feelings about family's household income when growing up: Getting by (ref: living comfortably)	**0.90**	**(0.86 - 0.95)**
[Childhood] Feelings about family's household income when growing up: Finding it difficult (ref: living comfortably)	**0.90**	**(0.84 - 0.97)**
[Childhood] Feelings about family's household income when growing up: Finding it very difficult (ref: living comfortably)	**0.83**	**(0.77 - 0.89)**
Country-level		
Proportion of population ages 65+ (std)	**0.51**	**(0.28 – 0.94)**
Proportion of female in labor force (std)	**1.79**	**(1.32 - 2.42)**
Fertility rate, total (births per woman) (std)	0.80	(0.50 - 1.29)
Proportion of religious composition (std)	0.78	(0.48 - 1.27)
Proportion of urban population (std)	0.84	(0.50 - 1.40)
Duration of compulsory education (std)	1.06	(0.78 - 1.43)
Unemployment rate (std)	1.06	(0.90 - 1.25)
Log GDP per capita	1.18	(0.71 - 1.96)
Gini coefficient (std)	1.29	(0.90 - 1.84)
Random part		
Variation in the interpret	0.30	(0.21 – 0.44)
Number of observations	131,487	
Number of countries	22	
Note: Bold values denote statistical significance at the $p<0.05$ level. The model was run on imputed data and was weighed.		

Table S8: Predicted probabilities from logistic regression models (based on imputed and weighted data)

	Ages 18-35 (vs ages 36-59)	Ages 60-99+ (vs ages 36-59)	Female (vs male)	With partner (vs no partner)	Number of children (aged under 18) in household	Religious attendance once a week+ (vs never)	Religious attendance once a month or less (vs never)	Living in city (vs rural/town)	Education: With degree (vs no degree)	Employment: Employed/ self-employed (vs retired)	Employment: Not employed/ other (vs retired)	Getting by (vs living comfortably)	Finding it difficult (vs living comfortably)	Finding it very difficult (vs living comfortably)	[Childhood] Getting by (vs living comfortably)	[Childhood] Finding it difficult (vs living comfortably)	[Childhood] Finding it very difficult (vs living comfortably)
**Argentina**	60.9%	-3.1%	-9.0%	0.7%	-3.8%	-5.6%	38.0%	-5.1%	109.9%	67.1%	30.7%	-1.3%	-27.1%	-58.7%	-22.1%	-31.0%	-69.0%
**Australia**	62.5%	-31.3%	67.4%	10.2%	-24.0%	74.5%	39.5%	33.1%	106.5%	65.9%	41.4%	-15.9%	-43.6%	-89.2%	18.1%	41.7%	-47.4%
**Brazil**	48.6%	-32.5%	-29.7%	-5.9%	-11.3%	56.0%	68.9%	19.8%	67.0%	-6.2%	-12.8%	-23.0%	-36.6%	-63.0%	-8.3%	-14.7%	-55.0%
**China**	-6.7%	-3.8%	4.9%	-14.7%	9.0%	119.7%	72.0%	-8.8%	37.6%	30.8%	4.6%	-22.7%	-55.9%	-82.1%	-12.4%	-37.6%	-37.1%
**Egypt**	52.5%	-116.2%	-45.9%	-86.5%	4.8%	52.6%	83.2%	-14.8%	86.3%	-80.3%	-78.7%	-53.1%	-28.7%	-67.9%	-20.7%	0.8%	-28.6%
**Germany**	72.7%	-4.7%	29.9%	-3.4%	8.0%	93.8%	68.6%	5.8%	93.9%	55.0%	69.3%	-10.5%	-35.0%	-60.0%	1.6%	23.2%	-10.2%
**Hong Kong**	103.9%	-65.6%	14.4%	54.7%	41.8%	141.3%	120.2%	44.5%	67.5%	-100.5%	-151.0%	-23.1%	-30.9%	-66.4%	-84.3%	-103.2%	-63.6%
**India**	8.3%	-27.1%	-57.5%	-34.0%	0.4%	31.2%	33.8%	-19.5%	45.8%	44.3%	59.5%	-1.2%	-3.4%	-3.3%	-27.7%	-28.8%	-21.5%
**Indonesia**	65.0%	-29.1%	-64.0%	-70.1%	2.7%	13.3%	20.7%	3.7%	96.7%	-0.3%	2.5%	5.3%	19.0%	-13.1%	-24.1%	-8.0%	-49.9%
**Israel**	27.0%	-24.1%	38.1%	-8.4%	-14.1%	-45.9%	5.0%	23.0%	63.7%	14.5%	-38.3%	7.7%	-15.8%	-48.8%	16.7%	-5.0%	-51.6%
**Japan**	23.7%	16.9%	41.6%	-18.8%	4.1%	113.9%	77.6%	26.3%	62.7%	-7.0%	-11.1%	-22.1%	-36.4%	-67.9%	-12.0%	-17.9%	-15.0%
**Kenya**	38.2%	-45.1%	-48.0%	-14.2%	1.6%	27.0%	18.4%	12.6%	39.7%	5.7%	4.4%	6.0%	6.3%	-19.7%	0.5%	-22.4%	-30.2%
**Mexico**	46.5%	-100.2%	-24.5%	-25.2%	17.1%	74.6%	80.2%	22.7%	103.9%	-27.4%	-50.6%	-43.6%	-70.3%	-91.1%	-37.8%	-60.0%	-78.6%
**Nigeria**	49.0%	-68.3%	-37.7%	12.0%	5.7%	35.0%	18.1%	27.4%	12.6%	-32.5%	-24.0%	9.0%	2.1%	-3.3%	5.3%	-9.0%	-26.8%
**Philippines**	17.7%	-19.3%	-33.0%	-38.5%	4.3%	34.1%	7.7%	-7.9%	0.0%	-10.2%	-24.4%	-22.4%	-28.4%	-28.4%	2.0%	-8.2%	-26.7%
**Poland**	30.9%	28.7%	29.1%	1.0%	7.6%	-48.2%	-28.1%	56.1%	44.3%	12.6%	45.0%	-36.7%	-14.4%	-68.2%	-24.1%	-6.9%	-19.2%
**South Africa**	23.2%	-88.0%	10.3%	-9.2%	1.5%	5.4%	7.2%	19.0%	63.2%	11.0%	25.4%	8.0%	9.1%	-6.7%	-20.0%	-24.6%	-4.5%
**Spain**	77.5%	-5.8%	34.3%	-7.9%	-10.4%	70.9%	22.1%	18.3%	78.5%	40.2%	-14.1%	-25.8%	-49.5%	-57.7%	-29.4%	-21.4%	14.0%
**Sweden**	-5.6%	21.5%	64.2%	-3.2%	-7.1%	81.8%	91.9%	31.4%	88.1%	2.2%	28.3%	-18.7%	-33.7%	-46.4%	2.7%	-0.3%	17.7%
**Tanzania**	41.1%	-24.3%	-72.0%	-41.1%	0.5%	79.4%	68.9%	-4.3%	54.0%	51.5%	46.1%	-3.9%	1.6%	-11.4%	-3.1%	-6.2%	-26.9%
**Türkiye**	62.1%	-126.6%	-54.3%	-38.0%	-14.6%	13.8%	1.3%	-4.0%	173.3%	-37.9%	-38.5%	-56.3%	9.4%	-83.9%	-1.9%	-51.9%	-73.2%
**United Kingdom**	29.4%	-31.5%	2.7%	6.9%	-1.4%	70.6%	76.5%	5.4%	121.9%	42.8%	-6.6%	-40.4%	-56.0%	-49.0%	-26.3%	-23.5%	-18.6%
**United States**	43.4%	-20.1%	50.6%	16.8%	-4.7%	45.6%	32.2%	17.7%	97.4%	9.5%	13.9%	-14.2%	-25.1%	-35.7%	-10.4%	-2.0%	-14.6%

Table note: These predicted probabilities demonstrate the relative importance of some individual-level factors above others. For example, in Hong Kong, the probability of engagement is on average 141.3 percentage points higher for people who had regular religious attendance, whereas in Türkiye, the probability is on average 13.8 percentage points higher.

Table S9: Imputation diagnostics.

	Imputation variance			RVI	FMI	Relative efficiency
	Within	Between	Total			
**Individual-level**						
Ages 18-35 (ref ages 36-59)	0.002304	6.30E-06	0.002312	0.003273	0.003268	0.999347
Ages 60-99+ (ref ages 36-59)	0.006581	4.00E-06	0.006585	0.000728	0.000728	0.999854
Female (ref male)	0.016252	4.60E-07	0.016252	0.000034	0.000034	0.999993
Marital status: Single (ref married)	0.004808	0.000015	0.004826	0.003693	0.003686	0.999263
Marital status: Separated/divorced/widowed (ref married)	0.002235	1.40E-06	0.002236	0.000769	0.000769	0.999846
Number of children aged under 18 in household	0.000175	4.90E-07	0.000176	0.003372	0.003366	0.999327
Frequency of religious attendance: Once a week or more (ref never)	0.005215	7.30E-06	0.005224	0.001678	0.001676	0.999665
Frequency of religious attendance: Once a month or less (ref never)	0.006793	1.10E-06	0.006794	0.000194	0.000194	0.999961
Living in city (ref rural/town)	0.002185	3.90E-06	0.002189	0.002138	0.002135	0.999573
Education: Secondary education (ref up to elementary)	0.004759	2.50E-06	0.004762	0.000628	0.000628	0.999875
Education: Beyond high school or college degree (ref up to elementary)	0.010071	2.50E-06	0.010074	0.000304	0.000303	0.999939
Employment status: Employed or self-employed (ref retired)	0.003689	3.00E-06	0.003692	0.000976	0.000975	0.999805
Employment status: Not employed or other (ref retired)	0.004464	2.10E-06	0.004467	0.000564	0.000563	0.999887
Feelings about household income: Getting by (ref living comfortably)	0.00063	3.30E-06	0.000634	0.00635	0.00633	0.998736
Feelings about household income: Finding it difficult (ref living comfortably)	0.001742	2.70E-06	0.001745	0.001859	0.001857	0.999629
Feelings about household income: Finding it very difficult (ref living comfortably)	0.004955	0.000011	0.004968	0.002678	0.002674	0.999465
[Childhood] Feelings about family's household income when growing up: Getting by (ref: living comfortably)	0.000685	4.20E-06	0.00069	0.007369	0.007341	0.998534
[Childhood] Feelings about family's household income when growing up: Finding it difficult (ref: living comfortably)	0.001384	1.80E-06	0.001386	0.001546	0.001545	0.999691
[Childhood] Feelings about family's household income when growing up: Finding it very difficult (ref: living comfortably)	0.001533	0.000016	0.001551	0.01216	0.012085	0.997589

Note: RVI=Relative Increases in Variance; FMI=Fraction of Missing Information.

Figure S1: Meta-analysis

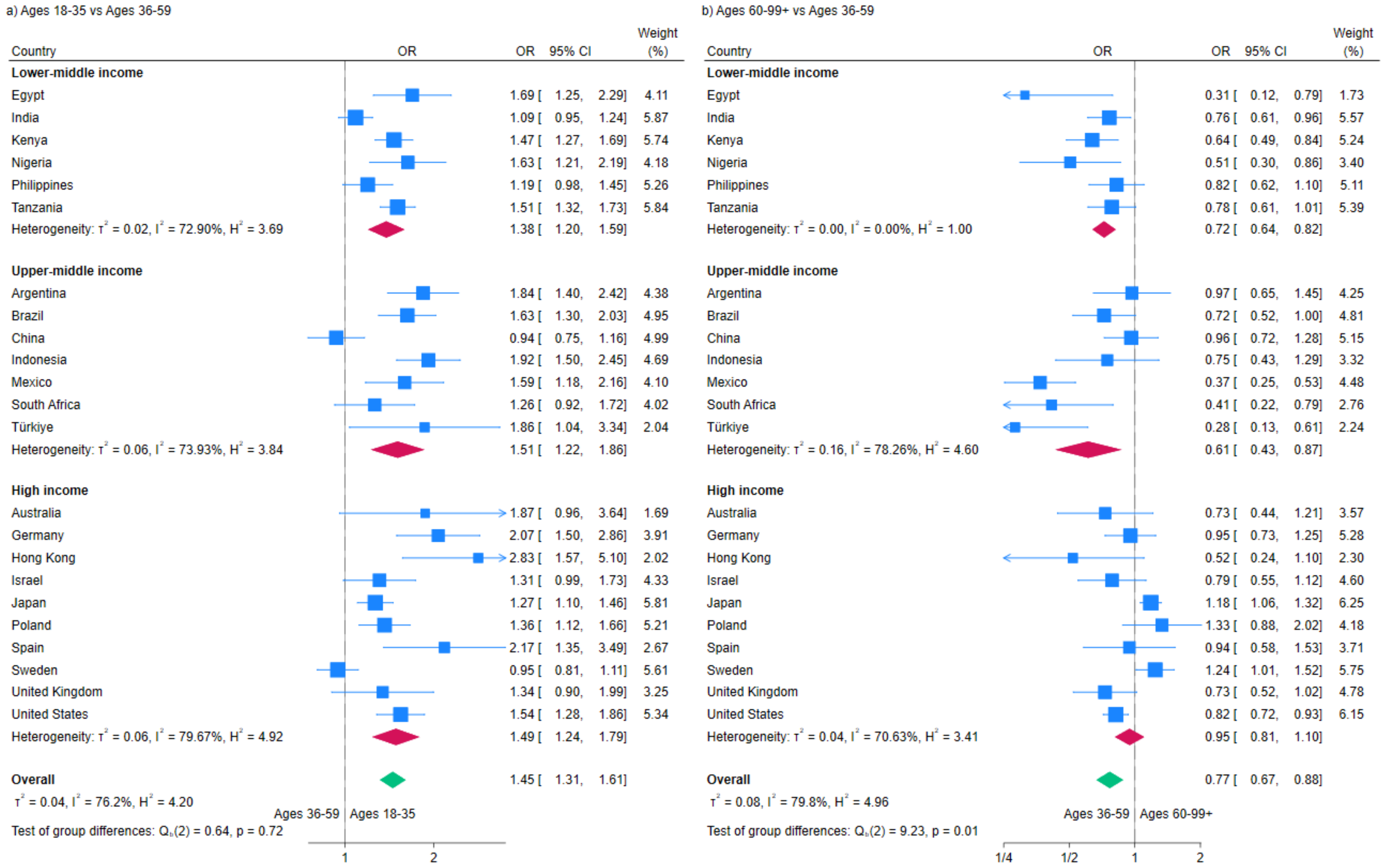

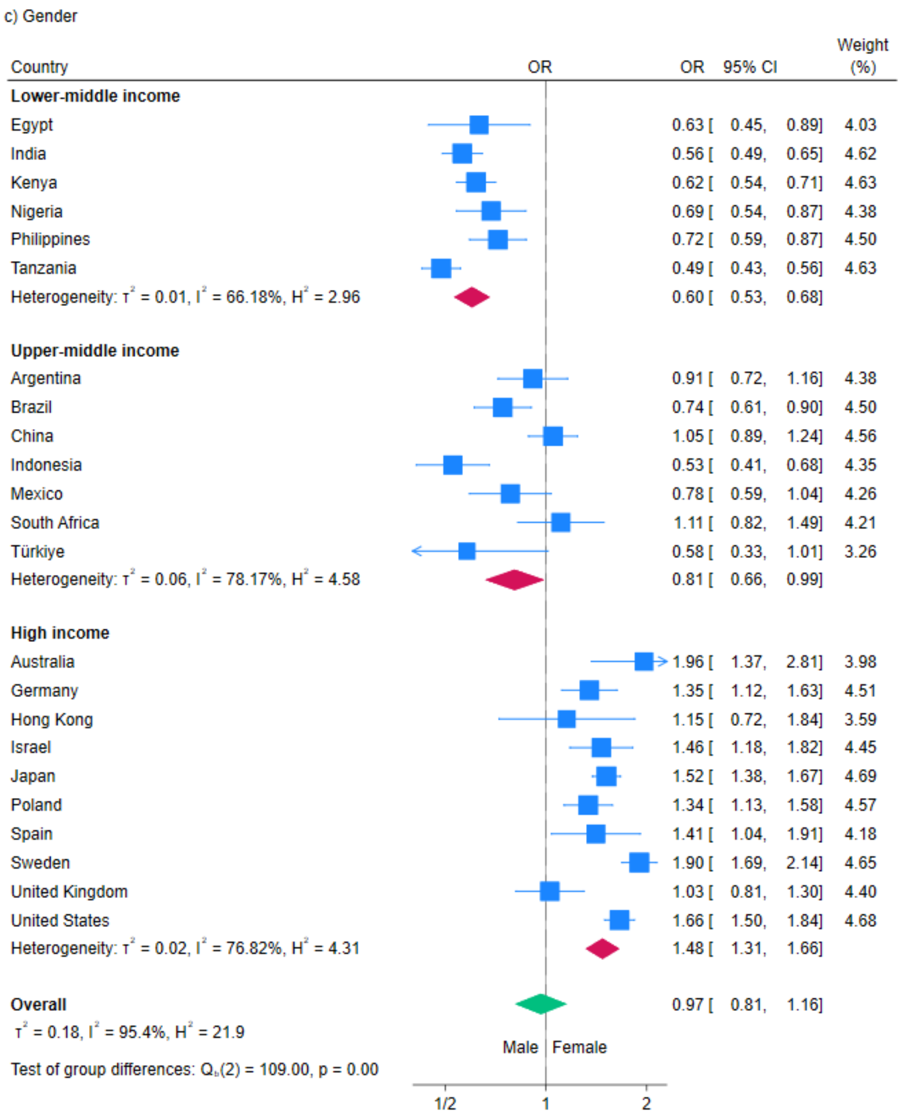
c) Gender
Country
OR
OR 95% CI
Weight (%)
Lower-middle income
Egypt 0.63 [0.45, 0.89] 4.03
India 0.56 [0.49, 0.65] 4.62
Kenya 0.62 [0.54, 0.71] 4.63
Nigeria 0.69 [0.54, 0.87] 4.38
Philippines 0.72 [0.59, 0.87] 4.50
Tanzania 0.49 [0.43, 0.56] 4.63
Heterogeneity: τ² = 0.01, I² = 66.18%, H² = 2.96 0.60 [0.53, 0.68]
Upper-middle income
Argentina 0.91 [0.72, 1.16] 4.38
Brazil 0.74 [0.61, 0.90] 4.50
China 1.05 [0.89, 1.24] 4.56
Indonesia 0.53 [0.41, 0.68] 4.35
Mexico 0.78 [0.59, 1.04] 4.26
South Africa 1.11 [0.82, 1.49] 4.21
Türkiye 0.58 [0.33, 1.01] 3.26
Heterogeneity: τ² = 0.06, I² = 78.17%, H² = 4.58 0.81 [0.66, 0.99]
High income
Australia 1.96 [1.37, 2.81] 3.98
Germany 1.35 [1.12, 1.63] 4.51
Hong Kong 1.15 [0.72, 1.84] 3.59
Israel 1.46 [1.18, 1.82] 4.45
Japan 1.52 [1.38, 1.67] 4.69
Poland 1.34 [1.13, 1.58] 4.57
Spain 1.41 [1.04, 1.91] 4.18
Sweden 1.90 [1.69, 2.14] 4.65
United Kingdom 1.03 [0.81, 1.30] 4.40
United States 1.66 [1.50, 1.84] 4.68
Heterogeneity: τ² = 0.02, I² = 76.82%, H² = 4.31 1.48 [1.31, 1.66]
Overall 0.97 [0.81, 1.16]
τ² = 0.18, I² = 95.4%, H² = 21.9
Male Female
Test of group differences: $Q_b(2)$ = 109.00, p = 0.00
1/2 1 2

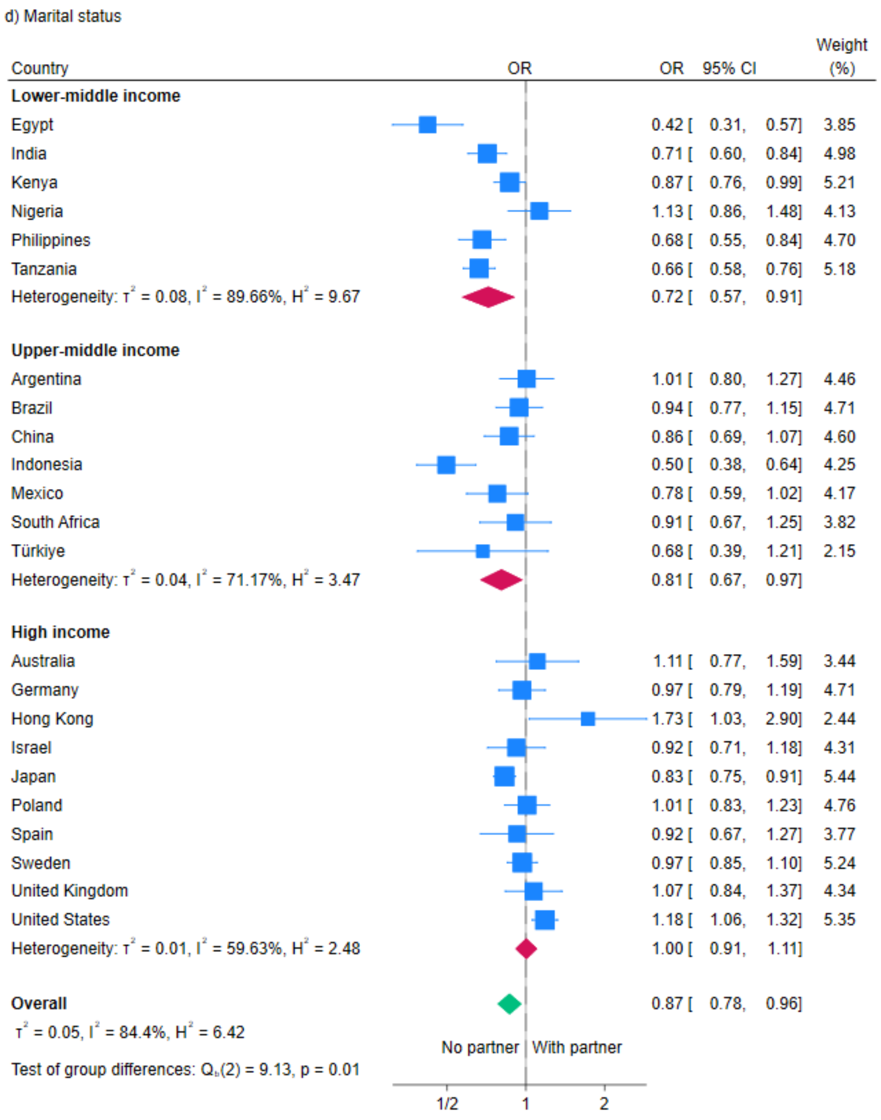
d) Marital status
Country
OR
OR 95% CI
Weight (%)
Lower-middle income
Egypt 0.42 [0.31, 0.57] 3.85
India 0.71 [0.60, 0.84] 4.98
Kenya 0.87 [0.76, 0.99] 5.21
Nigeria 1.13 [0.86, 1.48] 4.13
Philippines 0.68 [0.55, 0.84] 4.70
Tanzania 0.66 [0.58, 0.76] 5.18
Heterogeneity: τ² = 0.08, I² = 89.66%, H² = 9.67 0.72 [0.57, 0.91]
Upper-middle income
Argentina 1.01 [0.80, 1.27] 4.46
Brazil 0.94 [0.77, 1.15] 4.71
China 0.86 [0.69, 1.07] 4.60
Indonesia 0.50 [0.38, 0.64] 4.25
Mexico 0.78 [0.59, 1.02] 4.17
South Africa 0.91 [0.67, 1.25] 3.82
Türkiye 0.68 [0.39, 1.21] 2.15
Heterogeneity: τ² = 0.04, I² = 71.17%, H² = 3.47 0.81 [0.67, 0.97]
High income
Australia 1.11 [0.77, 1.59] 3.44
Germany 0.97 [0.79, 1.19] 4.71
Hong Kong 1.73 [1.03, 2.90] 2.44
Israel 0.92 [0.71, 1.18] 4.31
Japan 0.83 [0.75, 0.91] 5.44
Poland 1.01 [0.83, 1.23] 4.76
Spain 0.92 [0.67, 1.27] 3.77
Sweden 0.97 [0.85, 1.10] 5.24
United Kingdom 1.07 [0.84, 1.37] 4.34
United States 1.18 [1.06, 1.32] 5.35
Heterogeneity: τ² = 0.01, I² = 59.63%, H² = 2.48 1.00 [0.91, 1.11]
Overall 0.87 [0.78, 0.96]
τ² = 0.05, I² = 84.4%, H² = 6.42
No partner With partner
Test of group differences: $Q_b(2)$ = 9.13, p = 0.01
1/2 1 2

e) Number of children (aged under 18) in household

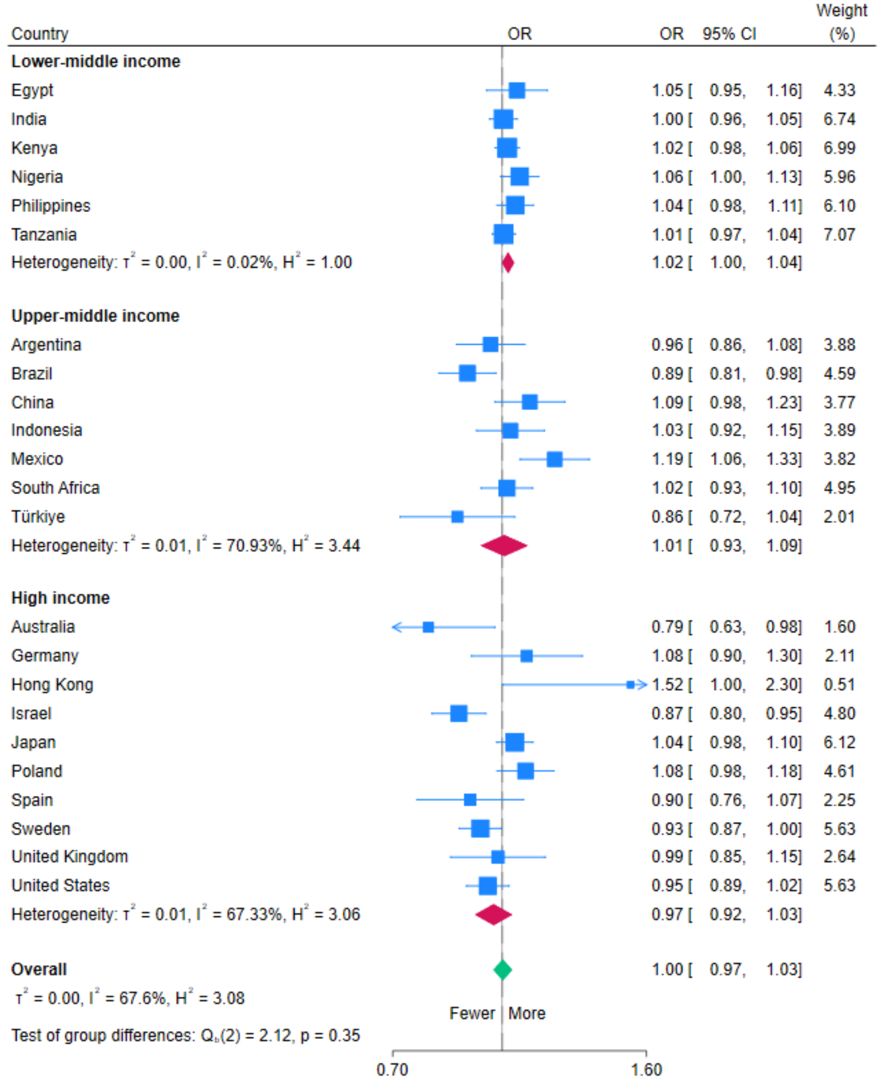

f) Religious attendance

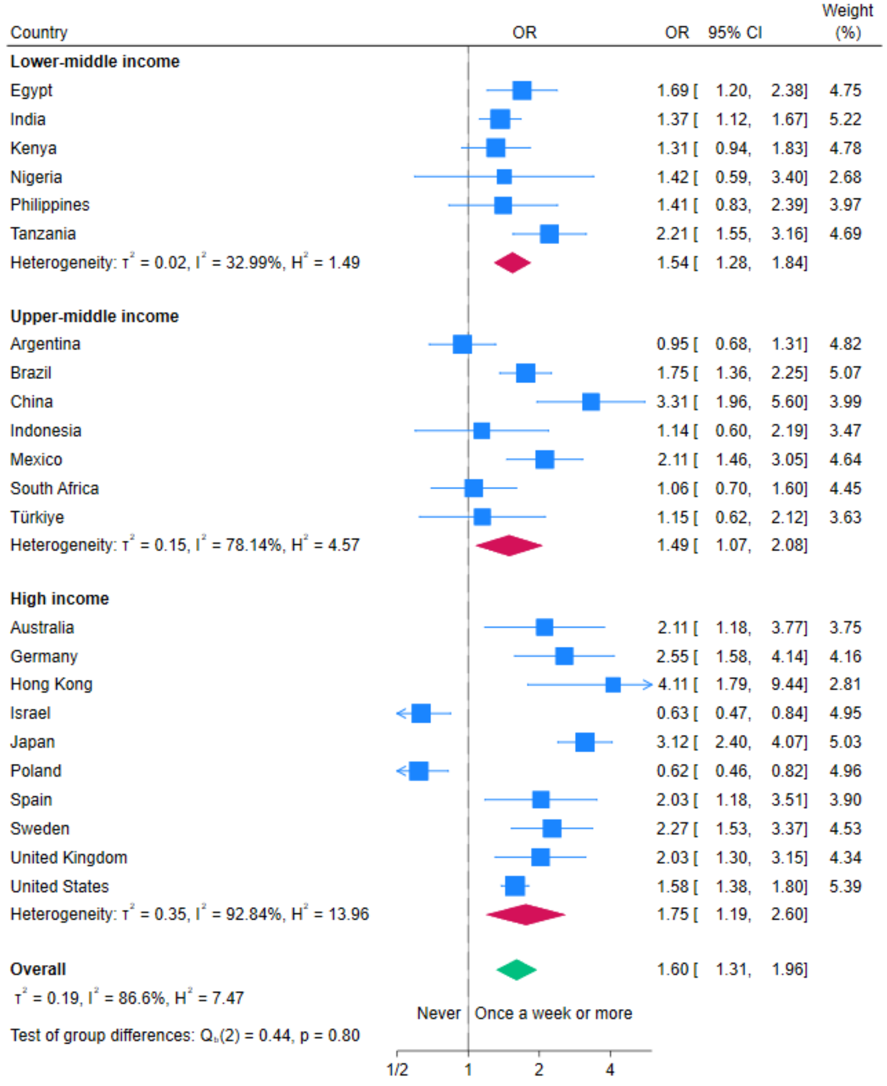

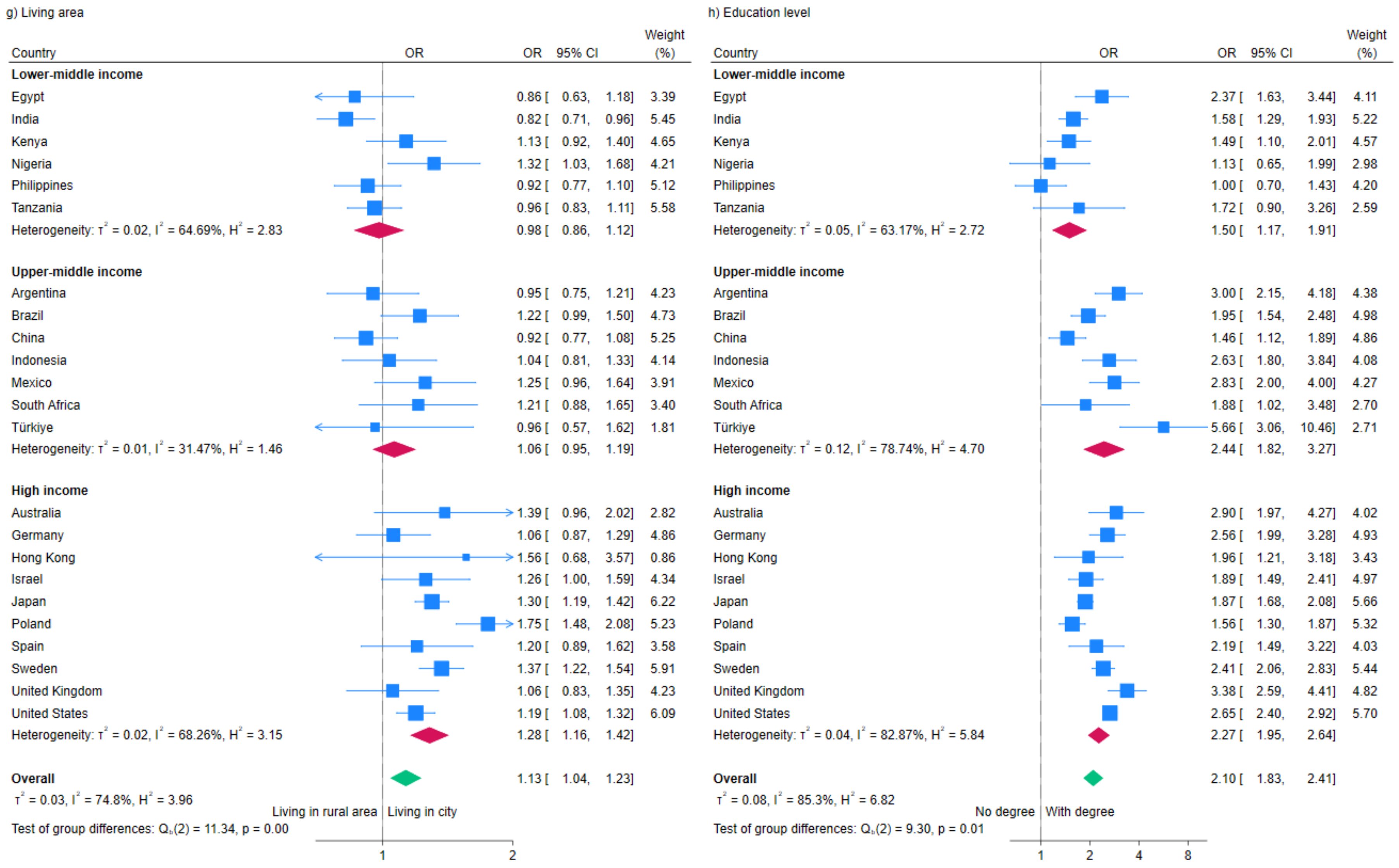
g) Living area
Country
OR
OR 95% CI
Weight (%)
Lower-middle income
Egypt 0.86 [0.63, 1.18] 3.39
India 0.82 [0.71, 0.96] 5.45
Kenya 1.13 [0.92, 1.40] 4.65
Nigeria 1.32 [1.03, 1.68] 4.21
Philippines 0.92 [0.77, 1.10] 5.12
Tanzania 0.96 [0.83, 1.11] 5.58
Heterogeneity: τ² = 0.02, I² = 64.69%, H² = 2.83 0.98 [0.86, 1.12]
Upper-middle income
Argentina 0.95 [0.75, 1.21] 4.23
Brazil 1.22 [0.99, 1.50] 4.73
China 0.92 [0.77, 1.08] 5.25
Indonesia 1.04 [0.81, 1.33] 4.14
Mexico 1.25 [0.96, 1.64] 3.91
South Africa 1.21 [0.88, 1.65] 3.40
Türkiye 0.96 [0.57, 1.62] 1.81
Heterogeneity: τ² = 0.01, I² = 31.47%, H² = 1.46 1.06 [0.95, 1.19]
High income
Australia 1.39 [0.96, 2.02] 2.82
Germany 1.06 [0.87, 1.29] 4.86
Hong Kong 1.56 [0.68, 3.57] 0.86
Israel 1.26 [1.00, 1.59] 4.34
Japan 1.30 [1.19, 1.42] 6.22
Poland 1.75 [1.48, 2.08] 5.23
Spain 1.20 [0.89, 1.62] 3.58
Sweden 1.37 [1.22, 1.54] 5.91
United Kingdom 1.06 [0.83, 1.35] 4.23
United States 1.19 [1.08, 1.32] 6.09
Heterogeneity: τ² = 0.02, I² = 68.26%, H² = 3.15 1.28 [1.16, 1.42]
Overall 1.13 [1.04, 1.23]
τ² = 0.03, I² = 74.8%, H² = 3.96
Living in rural area
Living in city
Test of group differences: Qb(2) = 11.34, p = 0.00
1
2
h) Education level
Country
OR
OR 95% CI
Weight (%)
Lower-middle income
Egypt 2.37 [1.63, 3.44] 4.11
India 1.58 [1.29, 1.93] 5.22
Kenya 1.49 [1.10, 2.01] 4.57
Nigeria 1.13 [0.65, 1.99] 2.98
Philippines 1.00 [0.70, 1.43] 4.20
Tanzania 1.72 [0.90, 3.26] 2.59
Heterogeneity: τ² = 0.05, I² = 63.17%, H² = 2.72 1.50 [1.17, 1.91]
Upper-middle income
Argentina 3.00 [2.15, 4.18] 4.38
Brazil 1.95 [1.54, 2.48] 4.98
China 1.46 [1.12, 1.89] 4.86
Indonesia 2.63 [1.80, 3.84] 4.08
Mexico 2.83 [2.00, 4.00] 4.27
South Africa 1.88 [1.02, 3.48] 2.70
Türkiye 5.66 [3.06, 10.46] 2.71
Heterogeneity: τ² = 0.12, I² = 78.74%, H² = 4.70 2.44 [1.82, 3.27]
High income
Australia 2.90 [1.97, 4.27] 4.02
Germany 2.56 [1.99, 3.28] 4.93
Hong Kong 1.96 [1.21, 3.18] 3.43
Israel 1.89 [1.49, 2.41] 4.97
Japan 1.87 [1.68, 2.08] 5.66
Poland 1.56 [1.30, 1.87] 5.32
Spain 2.19 [1.49, 3.22] 4.03
Sweden 2.41 [2.06, 2.83] 5.44
United Kingdom 3.38 [2.59, 4.41] 4.82
United States 2.65 [2.40, 2.92] 5.70
Heterogeneity: τ² = 0.04, I² = 82.87%, H² = 5.84 2.27 [1.95, 2.64]
Overall 2.10 [1.83, 2.41]
τ² = 0.08, I² = 85.3%, H² = 6.82
No degree
With degree
Test of group differences: Qb(2) = 9.30, p = 0.01
1
2
4
8

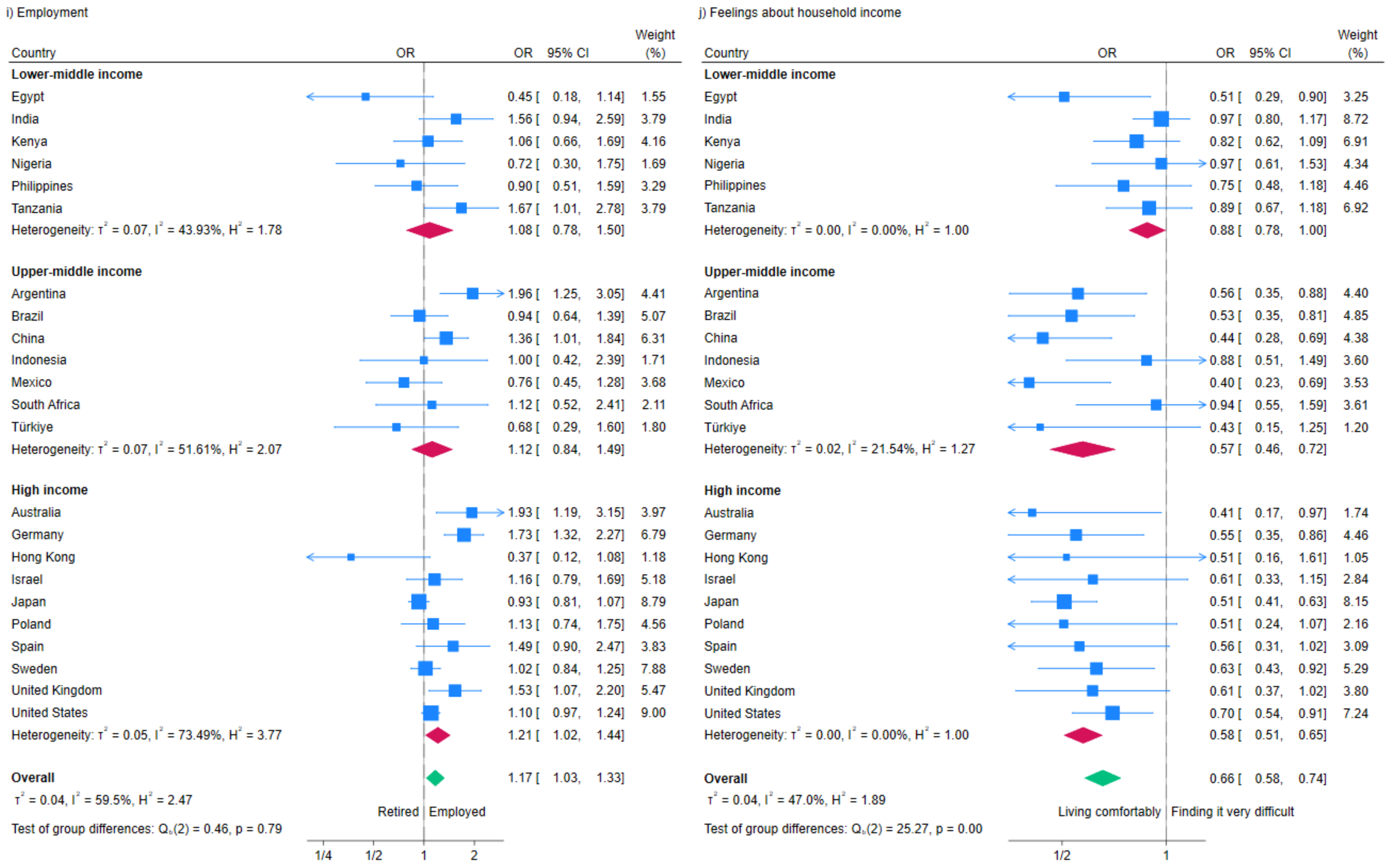

i) Employment
Country
OR
OR 95% CI
Weight (%)
Lower-middle income
Egypt 0.45 [0.18, 1.14] 1.55
India 1.56 [0.94, 2.59] 3.79
Kenya 1.06 [0.66, 1.69] 4.16
Nigeria 0.72 [0.30, 1.75] 1.69
Philippines 0.90 [0.51, 1.59] 3.29
Tanzania 1.67 [1.01, 2.78] 3.79
Heterogeneity: τ^2 = 0.07, I^2 = 43.93%, H^2 = 1.78 1.08 [0.78, 1.50]
Upper-middle income
Argentina 1.96 [1.25, 3.05] 4.41
Brazil 0.94 [0.64, 1.39] 5.07
China 1.36 [1.01, 1.84] 6.31
Indonesia 1.00 [0.42, 2.39] 1.71
Mexico 0.76 [0.45, 1.28] 3.68
South Africa 1.12 [0.52, 2.41] 2.11
Türkiye 0.68 [0.29, 1.60] 1.80
Heterogeneity: τ^2 = 0.07, I^2 = 51.61%, H^2 = 2.07 1.12 [0.84, 1.49]
High income
Australia 1.93 [1.19, 3.15] 3.97
Germany 1.73 [1.32, 2.27] 6.79
Hong Kong 0.37 [0.12, 1.08] 1.18
Israel 1.16 [0.79, 1.69] 5.18
Japan 0.93 [0.81, 1.07] 8.79
Poland 1.13 [0.74, 1.75] 4.56
Spain 1.49 [0.90, 2.47] 3.83
Sweden 1.02 [0.84, 1.25] 7.88
United Kingdom 1.53 [1.07, 2.20] 5.47
United States 1.10 [0.97, 1.24] 9.00
Heterogeneity: τ^2 = 0.05, I^2 = 73.49%, H^2 = 3.77 1.21 [1.02, 1.44]
Overall 1.17 [1.03, 1.33]
τ^2 = 0.04, I^2 = 59.5%, H^2 = 2.47
Test of group differences: $Q_b(2)$ = 0.46, p = 0.79
Retired | Employed
1/4 1/2 1 2
j) Feelings about household income
Country
OR
OR 95% CI
Weight (%)
Lower-middle income
Egypt 0.51 [0.29, 0.90] 3.25
India 0.97 [0.80, 1.17] 8.72
Kenya 0.82 [0.62, 1.09] 6.91
Nigeria 0.97 [0.61, 1.53] 4.34
Philippines 0.75 [0.48, 1.18] 4.46
Tanzania 0.89 [0.67, 1.18] 6.92
Heterogeneity: τ^2 = 0.00, I^2 = 0.00%, H^2 = 1.00 0.88 [0.78, 1.00]
Upper-middle income
Argentina 0.56 [0.35, 0.88] 4.40
Brazil 0.53 [0.35, 0.81] 4.85
China 0.44 [0.28, 0.69] 4.38
Indonesia 0.88 [0.51, 1.49] 3.60
Mexico 0.40 [0.23, 0.69] 3.53
South Africa 0.94 [0.55, 1.59] 3.61
Türkiye 0.43 [0.15, 1.25] 1.20
Heterogeneity: τ^2 = 0.02, I^2 = 21.54%, H^2 = 1.27 0.57 [0.46, 0.72]
High income
Australia 0.41 [0.17, 0.97] 1.74
Germany 0.55 [0.35, 0.86] 4.46
Hong Kong 0.51 [0.16, 1.61] 1.05
Israel 0.61 [0.33, 1.15] 2.84
Japan 0.51 [0.41, 0.63] 8.15
Poland 0.51 [0.24, 1.07] 2.16
Spain 0.56 [0.31, 1.02] 3.09
Sweden 0.63 [0.43, 0.92] 5.29
United Kingdom 0.61 [0.37, 1.02] 3.80
United States 0.70 [0.54, 0.91] 7.24
Heterogeneity: τ^2 = 0.00, I^2 = 0.00%, H^2 = 1.00 0.58 [0.51, 0.65]
Overall 0.66 [0.58, 0.74]
τ^2 = 0.04, I^2 = 47.0%, H^2 = 1.89
Test of group differences: $Q_b(2)$ = 25.27, p = 0.00
Living comfortably | Finding it very difficult
1/2 1

k) [Childhood] Feelings about household income

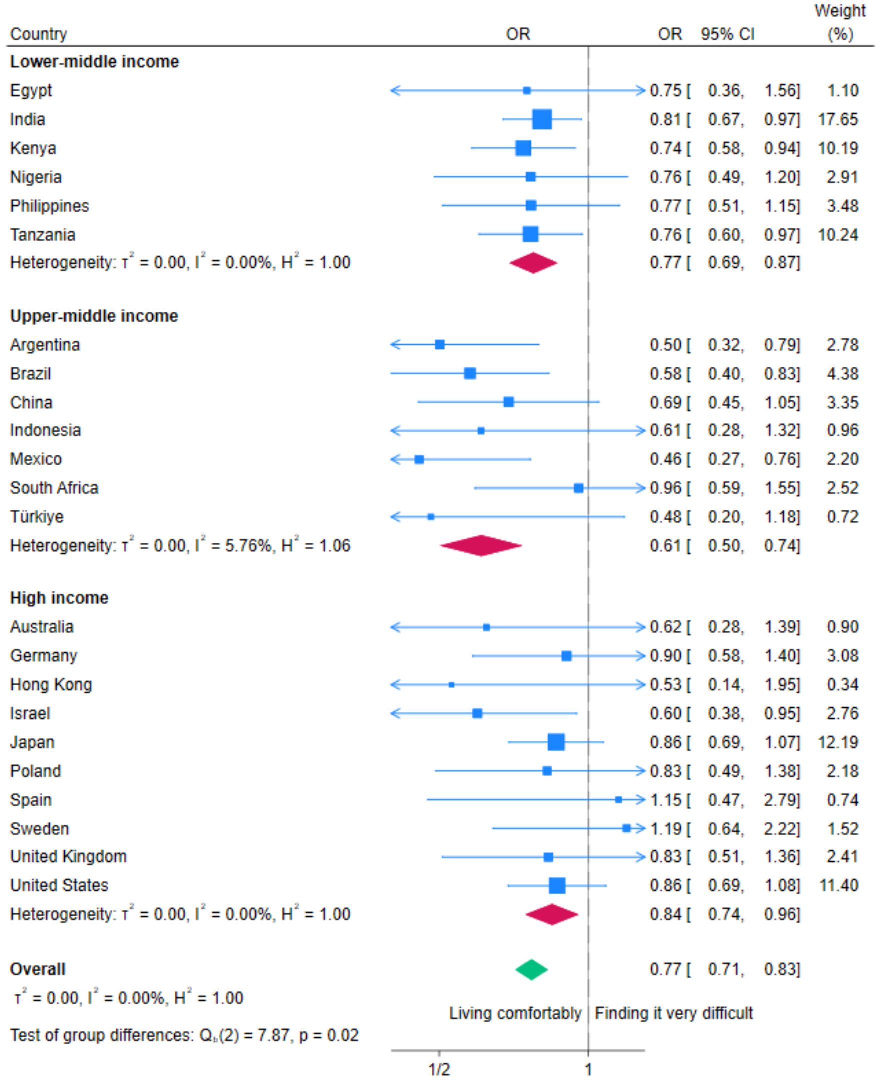

Figure S2: Sensitivity analysis – engaging monthly or more vs yearly

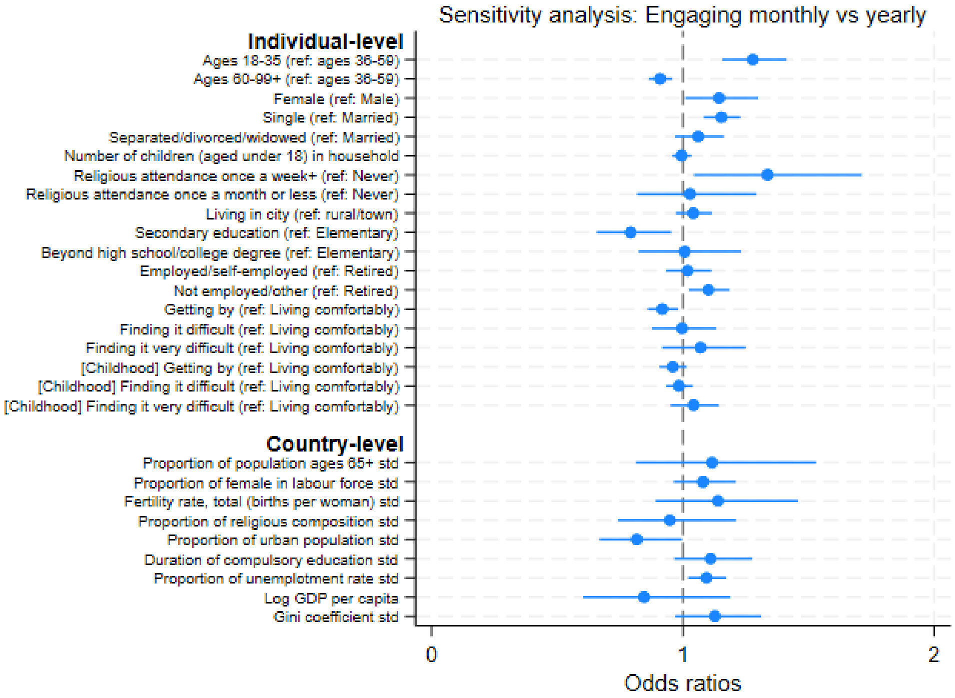

Figure S2 | Sensitivity Analysis- Multilevel modelling estimating the association between individual-level and country-level socio-economic factors and arts engagement (engaging monthly or more vs yearly). N=89,775 from 22 countries. *Individual-level demographic factors* included age, gender, marital status, numbers of children (aged under 18) in household, attendance frequency of religious services, living area, and education levels. *Economic factors* are employment status, feelings of their household income, and [childhood] feelings of their family's household income. *Country-level factors* included population ages 65+ (% of total population), female in labor force (% of total labor force), fertility rate: total (births per woman), religious composition %, urban population (% of total population), duration of compulsory education, unemployment rate (% of total labor force), logged GDP per capita, and Gini coefficient. All national predictors (except for GDP per capita) were standardized. The model was run on imputed and weighted data.

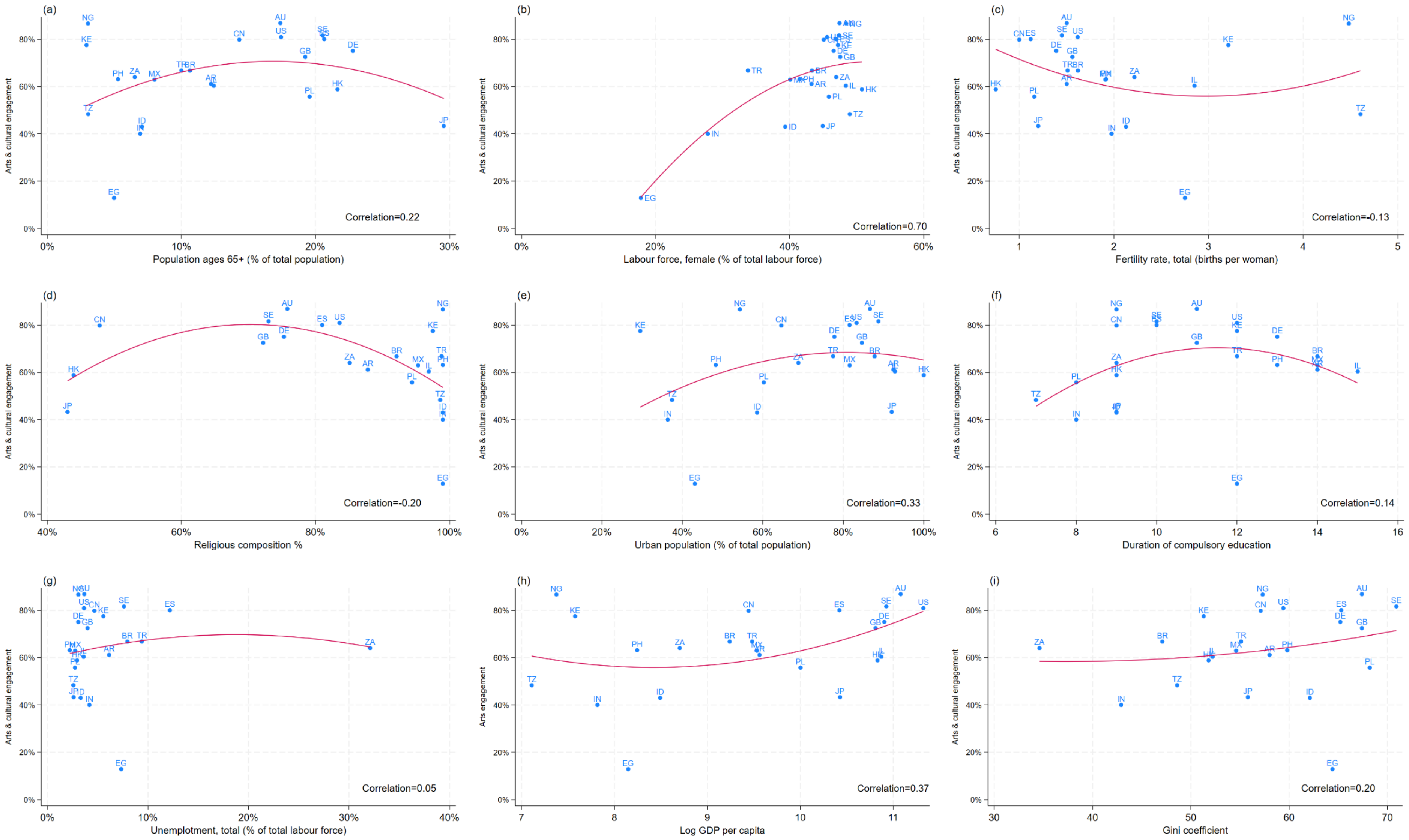

Figure S3 | Correlation between arts engagement and country-level factors. N=131,183 from 22 countries, based on unimputed and weighted data. a, Population ages 65+ (% of total population). b, Labor force, female (% of total labor force). c, Fertility rate total (births per woman). d, Religious composition %. e, Urban population (% of total population). f, Duration of compulsory education. g, Unemployment total (% of total labor force). h, log GDP per capita. i, Gini coefficient.

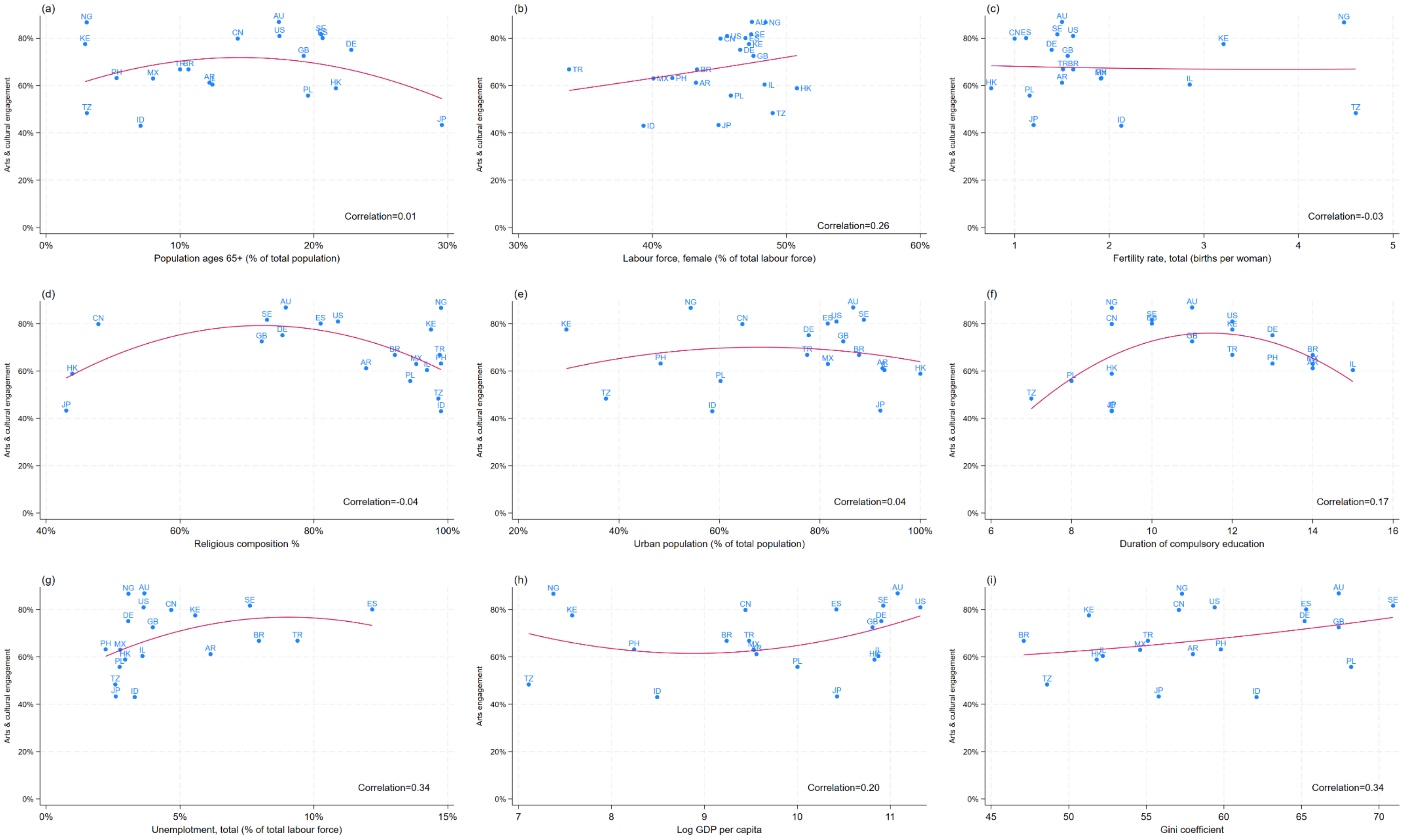

Figure S4: Correlation between arts engagement and country-level factors, removing South Africa, Egypt and India (N=131,183 from 19 countries, based on raw and weighted data). a, Population ages 65+ (% of total population). b, Labor force, female (% of total labor force). c, Fertility rate total (births per woman). d, Religious composition %. e, Urban population (% of total population). f, Duration of compulsory education. g, Unemployment total (% of total labor force). h, log GDP per capita. i, Gini coefficient.

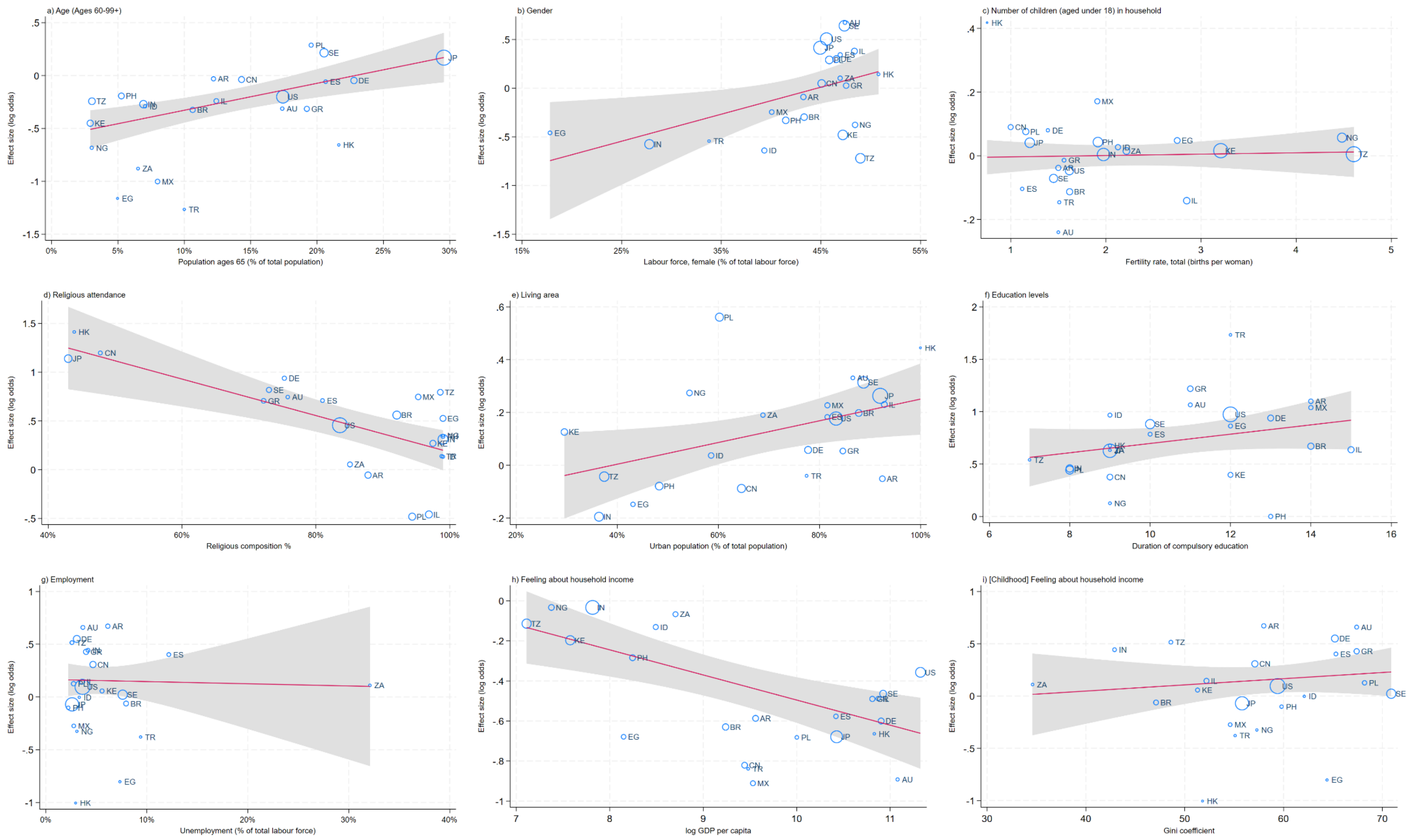

Figure S5: Meta-regression. Meta-regression was conducted to explore the heterogeneity variance in our meta-analysis using the nine country-level factors. For instance, in countries with higher proportions of women in the labor force, arts engagement was more common amongst women.
